# Parenting and Serious Mental Illness (SMI): A Systematic Review and Metasynthesis

**DOI:** 10.1007/s10567-023-00427-6

**Published:** 2023-02-18

**Authors:** C. I. Harries, D. M. Smith, L. Gregg, A. Wittkowski

**Affiliations:** 1 Division of Psychology and Mental Health, Faculty of Biology, Medicine and Health, School of Health Sciences, University of Manchester, 2Nd Floor Zochonis Building, Brunswick Street, Manchester, M13 9PL UK; 2 Greater Manchester Mental Health NHS Foundation Trust, Manchester, UK; 3 Manchester Academic Health Science Centre, Manchester, M13 9NQ UK

**Keywords:** Schizophrenia, Bipolar disorder, Psychosis, Mothers, Fathers, Service provision

## Abstract

The consequences of Serious Mental Illness (SMI) on parent and child outcomes can be profound. Supporting parents to manage their caregiving roles alongside parental SMI successfully has been recognised as a public health priority. To meet this priority and develop effective and acceptable interventions, it is imperative that parents’ experiences and support needs are understood. This systematic review aimed to synthesise qualitative research that explored parents’ experiences and perceptions of the impact of SMI on their parenting and their corresponding support needs. The Preferred Reporting Items for Systematic Review and Meta-Analysis guidelines were followed. Five databases were searched for terms associated with SMI, parenting, and qualitative research. Twenty-nine studies involving 562 parents who experienced SMI met inclusion criteria, and the methodological quality of included studies was appraised using the Critical Appraisal Skills Programme. After findings were synthesised using thematic synthesis, six themes were identified: (1) The constrained parent, (2) parenting difficulties, (3) the strained child, (4) inescapable threat, (5) combatting threat, and (6) wrap-around support needs. Novel insights into the centrality of SMI-related parenting difficulties and threat perceptions across parent, family, healthcare, and wider social systems on strained parent–child and distanced parent–support relationships were highlighted. Systemic practice change initiatives via compassionate and inclusive system-wide support were recommended.

**Supplementary Information:**

The online version contains supplementary material available at 10.1007/s10567-023-00427-6.

## Introduction

Parenting is complex and multifaceted, impacted by a multitude of personal and environmental factors. Around 4% of parents experience Serious Mental Illness^1^ (SMI; Stambaugh et al., 2017), such as psychosis, schizophrenia, and bipolar disorder, representing a large group of parents who face additional and often complex challenges when navigating parenthood (e.g. Dolman et al., 2013). Factors, such as social, emotional, and economic burden (e.g. Chen et al., 2021a, 2021b; Radley et al., 2022a, 2022b), have been associated with an increased risk of adverse outcomes in families within which a parent experiences SMI, including disrupted attachment relationships, social exclusion, child emotional difficulties, and parental suicidality (Dubreucq et al., 2021; Gregg et al., 2021; Perera et al., 2014). However, negative outcomes are not inevitable, and qualitative research suggests that parenting can offer a source of pride (Evenson et al., 2008), motivation, and hope for the future (Ackerson, 2003; Perera et al., 2014; Sabella et al., 2022). Despite this, much remains unknown about how parents living with SMI experience parenting and what their professional healthcare, practical, and peer support needs are in relation to parenting. In particular, parents’ experiences of navigating relationships with their children and wider systems, and their ideas about parenting support, are not yet fully understood. Consequently, parental needs are often disregarded by services, leaving parents feeling unheard and unsupported (David et al., 2011; Goodyear et al., 2022).

There has been an increase in research and policy guidance over the last 15 years focusing on the challenges that families face related to experiences of parental SMI (e.g. Bee et al., 2014; Diggins, 2011; Foster et al., 2019; Reedtz et al., 2021). Although positive advances have occurred, the focus has largely been on supporting children and other family members coping with the challenges of parental SMI and parental experiences have been largely neglected (Radley et al., 2022b). Major documents outlining practice change initiatives, including the National Health Service (NHS) Long-Term Plan (NHS England, 2019), fail to consider the support needs of parents who experience SMI adequately, despite policy guidance recommendations (e.g. Diggins, 2011). Furthermore, the inadequate implementation of family-focused practice (FFP) within countries that have legislation recommending its use (Furlong et al., 2021) highlights the insufficiency of policy-maker decisions alone in promoting practice change. Given that parent and child outcomes are inherently linked (Kahng et al., 2008), it is imperative that parental perceptions about the impact of SMI on parenting and their support needs are better understood to improve service provision for this priority group.

To better understand the experiences of mothers with SMI, Dolman et al. (2013) conducted a metasynthesis of 23 studies exploring 355 maternal experiences and eight studies exploring 143 healthcare professionals’ (HCPs) views. These studies, published between 1995 and 2011, reported on mothers’ experiences of preconception decision-making, pregnancy and motherhood with SMI, and HCPs experiences of providing support for these mothers. Guided by principles of metaethnography (Noblit & Hare, 1988), Dolman et al. (2013) identified two main themes, namely (1) experiences of motherhood and (2) experiences of services. However, the included studies are now more than a decade old, and the aims of that review were very broad: both pre- and post-conception experiences and mothers’ experiences of post-partum psychosis—a presentation with distinct clinical features that occurs in a discrete post-partum period (Spinelli, 2021)—were included, and the integral role of fathering was not considered. Thus, the applicability of findings to non-gendered SMI parenting experiences outside of the distinct perinatal period appears limited.

Prior to Dolman et al.’s (2013) review, Oyserman et al. (2000) reported a mixed review of 67 quantitative and qualitative studies published between 1980 and 2000. The authors reported that parental SMI was associated with disrupted attachment relationships and less attuned parenting. Although a relatively large number of studies were included, the included studies largely focused on mothers who experienced low mood or depression: only 9.3% of studies specified diagnoses of schizophrenia or psychosis and 9.7% of bipolar disorder. Moreover, a synthesis of included studies were not conducted which limits wider interpretations and conclusions from being drawn across studies. Other reviews have focused on parenting in the context of specific diagnostic characteristics. For example, in a mixed commentary of five quantitative and two qualitative studies published between 1969 and 2012, Engur (2017) reviewed parent ideas about the impact of experiences of psychosis on parenting. Engur (2017) found that parents experienced communication difficulties and disorganised parenting. However, little detail was provided, and the seven included studies were not synthesised. Similarly, other mixed reviews have limited their focus to specific presentations of bipolar disorder (Stapp et al., 2020), and qualitative reviews have restricted their focus to presentations of post-partum psychosis (Forde et al., 2020) or SMI in Chinese cultures (Chen et al., 2021a).

Although the reviews outlined above offer helpful insights into how parenting can be affected by specific diagnostic or cultural characteristics during discrete time periods, cross-cultural insights regarding the impact of SMI on parenting for both mothers and fathers, and their corresponding support needs, remain limited. To guide clinical practice for parents experiencing SMI effectively, an up-to-date and comprehensive qualitative understanding of how parenting is experienced in the context of SMI is required, in line with the Medical Research Council guidelines for the development of complex interventions (Skivington et al., 2021). Therefore, this metasynthesis aimed to synthesise parents’ experiences and perceptions of the impact of SMI on parenting to improve our understanding of their personal and professional support needs. We specifically addressed the question ‘What are parents’ experiences and perceptions of the impact of SMI on parenting and what support needs are indicated’? The outcomes of this metasynthesis can be used to inform policy, future research, and clinical practice.

## Methods

This metasynthesis was conducted in line with the Preferred Reporting Items for Systematic Reviews and Meta-Analysis (PRISMA) guidelines (Moher et al., 2009). The protocol was registered with PROSPERO on 01/12/2021 (Ref: CRD42021295443; https://www.crd.york.ac.uk/prospero/display_record.php?ID=CRD42021295443).

### Search Strategy

The search strategy was developed in consultation with the University of Manchester library service using the categories of Sample, Phenomenon of Interest, Design, Evaluation, and Research type from the SPIDER tool (Cooke et al., 2012; see Table 1). Medical Subject Heading (MeSH) terms were used to identify synonyms and Boolean operators (“AND”, “OR”) were used to combine terms and concepts. Five databases, relevant for this topic area, were searched: CINAHL, MEDLINE, EMBASE, PsychINFO and Web of Science. Databases were searched in December 2021 for articles published from inception that contained the terms outlined in Table 1, either in the title, abstract, or keywords. The search was updated in April 2022 which identified one new study for inclusion. Google Scholar and reference lists of included studies were searched (Horsley et al., 2011).

**Table 1:** Search terms and limits

1.	S-ample	(Parent* or Mother* or Father* or Caregiv* Guardian* or Carer* or Kinship or Stepparent* or foster parent*)
2.	PI-phenomenon of Interest	(Serious Mental Illness* or SMI or Severe Mental Illness* or Enduring Mental Illness* or Serious Mental Health Difficult* or Serious Mental Health Problem* or Psychos* or Schizophr* or Mental Health or Mental Illness* or Persistent Mental Illness* or Bipolar* Disorder* or Bipolar*)
3.	D-design	(interview*, focus group*, case stud* or observ*)
4.	E-evaluation	(view* or experience* or opinion* or attitude* or perce* or belie* or feel* or know* or understand* or Perspective*)
5.	R-research type	(Qualitative* or mixed method* or IPA or Grounded Theory or Thematic Analys* or Narrative*)
6.	1 AND 2	
7.	3 OR 4	
8.	5 AND 6 AND 7	

Limits: Human, Peer-reviewed, English, and German language

**Fig. 1:**
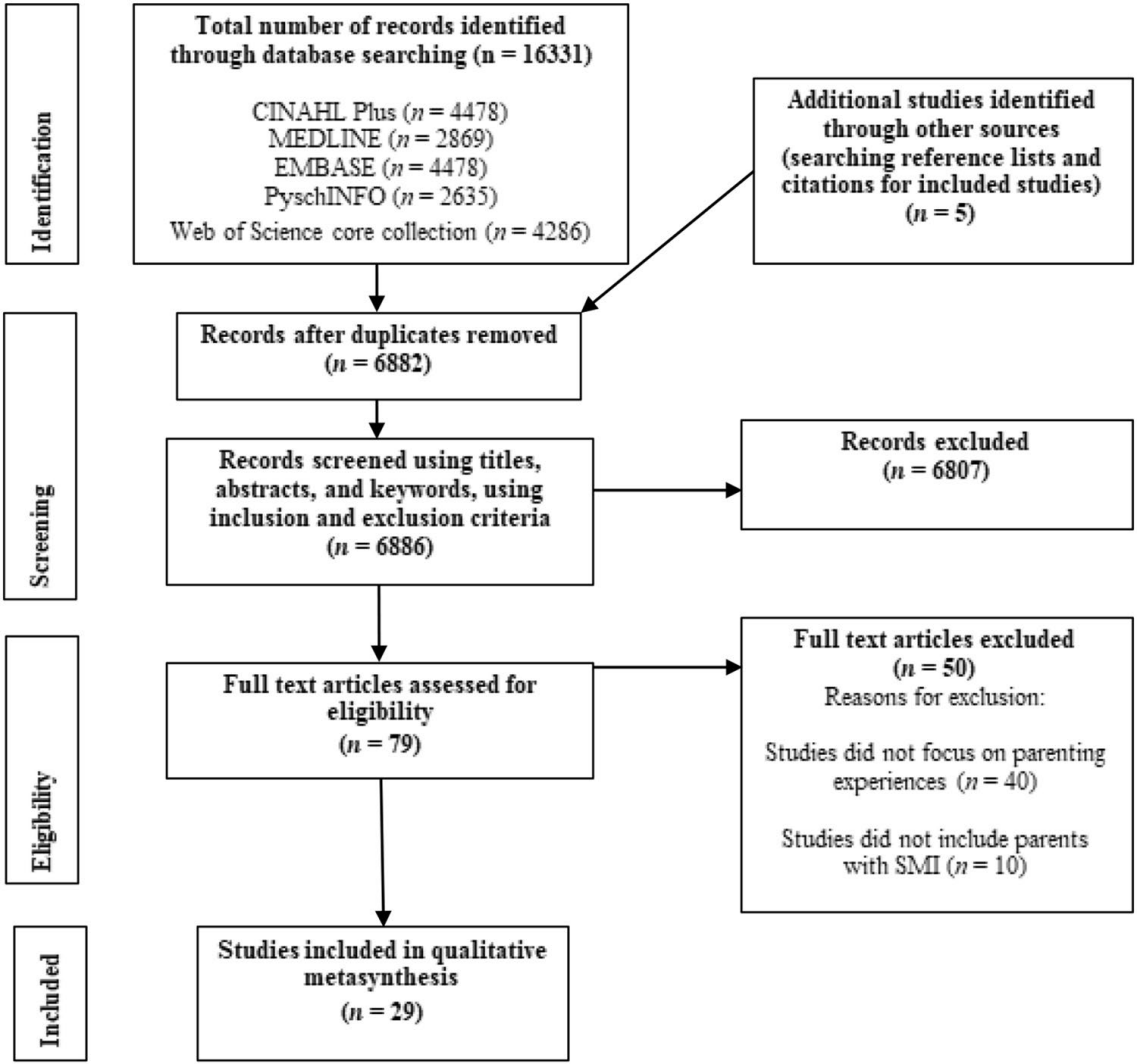
PRISMA flow diagram

An outline of the systematic search process is illustrated in Fig. 1. Identified references were imported into EndNote (Clarivate Analytics UK Ltd [Version 20], 2020). Duplicates were removed, and titles, keywords, and abstracts were assessed for eligibility against the inclusion and exclusion criteria by the first author. A second independent reviewer assessed a sample of 10% (*n* = *688*) of the total number of studies for inclusion (*N* = *6881*). Agreement between reviewers was substantial (99.85%, κ = 0.67) and any discrepancies were resolved through discussion. The first author reviewed the full text of studies that were not excluded during the screening stage. In the instance of uncertainty, two other authors jointly reviewed the studies and discussions were held to reach agreement. Corresponding authors of included studies were contacted via email regarding data queries when diagnostic information pertaining to study samples were not presented.

### Inclusion and Exclusion Criteria

Papers were included if they (1) were written in English or German (as the research team was fluent in these languages), (2) included qualitative data from qualitative or mixed methods studies that could be extracted, (3) involved parents (mothers, fathers, stepparents, guardians, adoptive parents, foster parents, or kinship parents) who experienced SMI defined as schizophrenia-spectrum disorders, psychosis, or bipolar disorder not limited to the perinatal period (conception to the child’s second birthday), (4) focused on parenting experiences, and (5) were published in a peer-reviewed journal. Papers were excluded if they focused on parenting in the perinatal period only or exclusively included parents who no longer had contact with their children.

### Methodological Quality and Risk of Bias Assessment

Each included study’s methodological quality/risk of bias was appraised using the widely used 10-item Critical Appraisal Skills Programme (CASP, 2018) for qualitative research. As the CASP does not offer a summary scoring system (Long et al., 2020), a numerical system was used (No = 0, Partially Agree = 0.5, Yes = 1). Methodological quality was categorised as high (> 8–10), moderate (6–8), or low (≤ 5; see Butler et al., 2020). To ensure reliability of assessment ratings, another independent reviewer rated 100% of the included papers. Substantial agreement was achieved between reviewers (96.32%, *κ* = 0.76). Any disagreements were resolved by discussion.

### Data Extraction and Analysis

Quotations from parents within included studies under the headings ‘results’ or ‘findings’ were extracted into Microsoft Word and analysed using Thomas and Harden’s (2008) thematic synthesis. Author interpretations from the included studies were also extracted to inform the analysis; however, theme development was concentrated on parent quotations. This approach promotes the integration of qualitative findings from multiple studies via the identification of common themes across studies. The approach has been identified as promoting consideration of the appropriateness and acceptability of service provision (Barnett-Page & Thomas, 2009), thus allowing policy and practice to be informed.

The synthesis followed three overlapping stages (Thomas & Harden, 2008). The first author led on all stages and began by reading each included study several times before line-by-line coding the extracted data using pen and paper methods, from which 1840 preliminary codes were developed. Next, descriptive themes were developed inductively across papers using ‘post-it’ notes. Sub-themes and analytical themes were developed in the final stage by interpreting consistent and inconsistent themes across papers, relying on researcher inference and judgement. The use of quotations from parents has been indicated throughout the text using quotation marks.

To minimise potential bias, another author independently analysed five of the included studies, which were randomly selected. The research team discussed the analytical themes to ensure that the final themes were plausible, coherent, and appropriately derived from the data. A critical realist epistemology underpinned the analysis (Fletcher, 2017), allowing for inferences about psychosocial processes around parenting to be made, whilst recognising that inferences are bound by the context of the research that psychosocial phenomena can exist independently of theory, but that meaning can be constructed from the experiences reported within the included studies. Enhancing Transparency in Reporting the synthesis of Qualitative research (ENTREQ) guidelines was followed (Tong et al., 2012; Supplementary Material 1).

### Reflexivity Statement

The authors were all white European women who ranged in ages and three were parents. The first author was a trainee clinical psychologist with several years of experience working in clinical and research roles with people experiencing SMI. The second author was an academic psychologist specialising in health psychology research with an interest in parenting. The third author was an academic psychologist specialising in psychosis research with an interest in family-focused practices. The fourth author was an academic and clinical psychologist with an interest in understanding and supporting mothers who experience SMI. As a team, we were conscious of evaluating the extracted data from a clinical research and health perspective. We were aware that the parents within the included studies were more likely to have experienced socio-economic disadvantage and we were conscious of power differentials that could have existed between health professionals, researchers, and service users (Johnstone & Boyle, 2018). As a team, we tried to be aware of not biasing interpretations towards a white euro-centric viewpoint. A reflective diary, research team discussions, and a rigorous research process were utilised to minimise the potential for biased interpretations of parental quotes and author interpretations.

## Results

### Study Characteristics

Twenty-nine studies were identified and synthesised (see Fig. 1). These studies were conducted in 14 countries between 1995 and 2022 and reported on the parenting experiences of 562 mothers and fathers who experienced SMI (see Table 2). Most studies reported mothers’ experiences (*n* = 16), 12 reported mothers’ and fathers’ experiences, and one study reported fathers’ experiences only (Evenson et al., 2008). Most studies detailed participant age, sex, and number of children, but few other socio-economic demographical characteristics were reported. For example, only nine of the 29 studies (31.03%) reported on the ethnicity of participants. Sample sizes ranged from five to 57. Of the 26 (89.65%) studies that detailed diagnoses, 46.86% of participants were reported to have diagnoses of schizophrenia or psychosis and 21.41% of bipolar disorder. Diagnoses were verified by self-report (*n* = 4), the Diagnostic and Statistical Manual of mental disorders, fourth edition (DSM-IV; American Psychiatric Association [APA], 1994; *n* = 1), and the DSM-IV-text revised (DSM-IV-TR; APA, 2000; *n* = 2) or the International Classification of Diseases, 10th edition (ICD-10; World Health Organisation [WHO], 1993; *n* = 2). However, 20 studies did not state how diagnoses were verified clearly. Qualitative data were derived from interviews (*n* = 28) or focus groups (*n* = 1), and a range of analysis methods were used.

**Table 2:** Characteristics of included studies presented in chronological order

	Study: Authors, Year, Location	Study Aim	Sample Description			Verification of diagnosis	Recruitment method	Data collection^a^/analysis	Main theme titles
			Parental mental health difficulty	Other socio-demographical information	Child information				
1.	Radley et al., (2022a), UK	To understand the needs and experiences of parents with psychosis	Psychosis (*n* = 12; 100%)	Mothers (*n* = 10) and fathers (*n* = 2) Marital status: married (*n* = 6), divorced (*n* = 1), separated (*n* = 1), single (*n* = 4) Ethnicity: White British (*n* = 7), White(other) (*n* = 1), Black British (*n* = 3), Asian British-Pakistani (*n* = 1) Age (years): 20–29 (*n* = 1), 30–39 (*n* = 5), 40–49 (*n* = 4), 50–59 (*n* = 2) Years since first psychotic episode ranged from 0 to 33 No. of hospital admissions due to psychosis ranged from 0 to 4 Setting: community	No. of children: 1 (*n* = 6) 2 (*n* = 3) 3 (*n* = 3) Child genders: M (16), F(5)	Not stated	Early Intervention in Psychosis services and Adult mental health teams	Semi-structured interviews / Reflexive TA (Braun & Clarke, 2006, 2020)	- The impact of psychosis on parenting - The need to protect their child - The need to feel normal - The impact of parenting stress on psychosis
2.	Sabella et al. (2022), USA	To understand the experiences of young adult parents with SMI	Anxiety (*n* = 15; 28%), MDD (*n* = 13; 24%), PTSD (*n* = 9; 17%), BD (*n* = 9; 17%), schizophrenia (*n* = 1; < 1%), schizoaffective disorder (*n* = 1; < 1%), ED (*n* = 5; 9%), BPD (*n* = 1; < 1%)	Mothers (*n* = 15) and fathers (*n* = 3) Age (years): Mean age 26 Ethnicity: non-Hispanic white (*n* = 10), non-Hispanic black (*n* = 2), Hispanic (*n* = 3), mixed/other (*n* = 3) Relationship status: married/ cohabiting (*n* = 5), divorced/separated (*n* = 3), never married (*n* = 10) Living: independently (*n* = 9), with own parents (*n* = 6), homeless shelter/group home (*n* = 3) Income: average income < $10,000 Education: high school (*n* = 8), college (*n* = 7), degree (*n* = 3) Setting: community	Number of children: 1 (*n* = 9), 2 (*n* = 7), 3 (*n* = 1), 4 (*n* = 1). Ages ranged from “several weeks” to 12 years	Unclear	Community centres, social services, homeless shelters, and social media advertisement	Semi-structured interviews / GT (Strauss & Corbin, 1998)	- Managing symptomatology whilst parenting - Children as sources of motivation and recovery - Experiences of discrimination and feelings of stigma
3.	Chen et al., (2021b), China	Explore the experiences of family life and parenting of Chinese mothers, in the context of mental illness	Schizophrenia (*n* = 1; 7%), BD (*n* = 3; 21%), Anxiety (*n* = 2; 14%), MDD (*n* = 4; 28%), PND (*n* = 3; 21%), Anxiety and Depression (*n* = 1; 7%)	Mothers (*N* = 14) Age (years): 20–29 (*n* = 1), 3–39 (*n* = 8), 40–49 (*n* = 5) Relationship status: Married (*n* = 12), divorced (*n* = 1), new relationship (*n* = 1) Employment: employed (*n* = 11), part-time (*n* = 1), freelance (*n* = 1), housewife (*n* = 1) Ethnicity: Chinese (*n* = 14) SES status: not reported Setting: outpatient	Number of children: 1 (*n* = 9), 2 (*n* = 5) Aged 1–6 (*n* = 5), 7–12 (*n* = 10), 13–18 (*n* = 4)	Self-report	Social media	Semi-structured interviews / IPA (Smith et al., 2009)	- Motherhood as central identity - The stigma associated with being a mother with MI - The impact of MI on parenting - Perceptions about the impact of MI on children - Experience of talking to children about MI - How having children impacts mothers’ MI and their recovery - Support obtained and needed
4.	Mulvey et al. (2021), USA	To explore how mothers involved in the criminal justice system with significant and long-term mental illness describe their experience of mothering	Bipolar/depression type disorder (*n* = 31; 65%), psychotic disorder (*n* = 14; 29%) and anxiety type disorder (*n* = 3; 6%)	Mothers (*N* = 48) Age (years): Mean age 40 Ethnicity: from white (*n* = 30), black (*n* = 10), Hispanic (*n* = 4), or other (*n* = 4) backgrounds Custody: never lost custody (*n* = 18), partial/family custody (*n* = 11), and lost custody at some point (*n* = 19) SES: not reported Setting: community	The number of children participants had 1 (*n* = 12), 2 (*n* = 13), 3 (*n* = 10), or 4 or more (*n* = 13)	DSM-IV-TR diagnoses	SMI probation caseloads within the criminal justice system	Semi-structured interviews / Unspecified inductive approach inspired by GT	- “Normative” Mothering - Aspiring to Break the Cycle - Constrained Mothering - “Failure” and State Intervention - Children as Parents - Children as Catalyst for Change
5.	Boström and Strand (2021), Sweden	To explore parent–infant relationships and parent and child mental health perceptions	Schizoaffective disorder (*n* = 4; 67%) and schizophrenia (*n* = 2; 33%)	Mothers (*n* = 4) and fathers (*n* = 2) Age (years): 38–47 Family environment: cohabiting with other parent (*n* = 2), single parent (*n* = 1), shared custody (*n* = 2), child in foster care (*n* = 1) Ethnicity and SES: not reported Setting: community	Children: five girls and two boys aged 8–15	Not stated	Four outpatient services for people who experience psychosis	Semi-structured interviews / IPA (Smith et al., 2009)	- An unclear image - An incoherent story - Illness as part of ordinary life - A non-hierarchical parent–child relationship - Attunement of the parent–child relationship and child well-being
6.	Strand et al. (2020), Sweden	To explore parents’ experiences of how psychosis affects their parenting	Schizoaffective disorder (*n* = 8; 53%), schizophrenia (*n* = 2; 13%), psychotic disorder (*n* = 3; 20%) and MDD with psychotic episodes (*n* = 2; 13%)	Mothers (*n* = 10) and fathers (*n* = 5) Age (years): 36–56 (*M* = 42) Relationship status: married/cohabiting (*n* = 8), single, divorced (*n* = 7) Employment: employed (*n* = 2), parental leave (*n* = 1), sick leave (*n* = 12) Custody status: lived with child/ren (*n* = 10), joint custody (*n* = 3), access rights (*n* = 2). Ethnicity and SES: not reported Setting: community	Participants had 17 children between them (11 boys and 6 girls), aged 3–16 (*M* = 10)	Not stated	Psychosis outpatient clinics	Semi-structured interviews / TA (Braun & Clarke, 2006)	- Protection - Reciprocity - Control - Guided learning - Group participation - Unpredictable absences
7.	Chan et al. (2019), Hong Kong	To explore the experiences of parenting and self-stigmatisation of Chinese mothers with SMI	MDD (*n* = 11; 73%) or a schizophrenia-spectrum disorder (*n* = 4; 27%)	Mothers (*N* = 15) Age (years): 26—50 Relationship status: divorced (*n* = 8), married (*n* = 5), windowed (*n* = 2) Employment status: unemployed (*n* = 10), part-time (*n* = 4), full-time (*n* = 1) Ethnicity and SES not reported Setting: community	Participants had between 1 and 6 children between them, aged between two and 22 years	Not stated	Two mental health support centres	Semi-structured interviews / TA (Braun & Clarke, 2006)	- Distancing and being distanced - Doubting myself - Struggling for control
8.	Awram et al. (2017), Australia	To understand the strategies women with mental illness use to balance the demands of mothering with mental health recovery	Depression (*n* = 6; 29%), BD (*n* = 3; 14%), schizoaffective disorder (*n* = 2), PND (*n* = 1; 10%), post-natal psychosis (*n* = 1; 5%), anxiety (*n* = 4; 20%), PTSD (*n* = 3; 14%), OCD (*n* = 1; 5%)	Mothers (*N* = 10) Relationship status: separated (*n* = 4) living with partner (*n* = 6), Childcare arrangements: children in mothers’ full-time care (*n* = 6), part-time care (*n* = 3), or both (*n* = 1) Ethnicity and SES status not reported Setting: community	Mothers had between 1 and 4 children aged 2–25 years (*M* = 12)	Not stated	Three community mental health organisations	Semi-structured interviews / GT and constant comparison (Charmaz, 2014)	- Recovery and motherhood intertwined - Seeing the bigger picture - Strategies of balancing mothering and recovery - Supports and resources
9.	Klausen et al. (2016), Norway	To understand mothers’ stories about motherhood in relation to being admitted as mental health service users	Psychosis (*n* = 2; 20%), suicidality (*n* = 2; 20%), somatic illness (*n* = 1; 10%), overdose (*n* = 1; 10%), depression (*n* = 1; 10%), “request from doctor” (*n* = 1; 10%), not disclosed (*n* = 2; 10%)	Mothers (*N* = 10) Age (years): 31–70 Relationship status: single (*n* = 3), married (*n* = 3), had a partner (*n* = 4) Ethnicity and SES not reported Setting: community perspective on inpatient admission	The number of children participants had 2 (*n* = 5), 3 (*n* = 3), 4 (*n* = 1), and 6 (*n* = 1). Age of children not specified	Unclear	Psychiatric hospital services	Semi-structured interviews / TA (Riessman, 2008)	- Being able to put oneself in the child’s shoes - The emotional impact of being admitted - Being open with the children about the admission - Being an emotionally available and present mother
10.	van der Ende et al. (2016), Netherlands	To understand the successful strategies of parents with mental illness	Mood disorder (*n* = 9; 33%), anxiety (*n* = 1; 4%), psychotic disorder (*n* = 6; 22%), addiction (*n* = 2; 7%), PD (*n* = 7; 26%), and ADHD (*n* = 2; 7%)	Mothers (*n* = 19) and fathers (*n* = 8) Age (years): 19–59 Relationship status: married/ relationship (*n* = 14), divorced/ widowed (*n* = 5), unmarried (*n* = 8) Living arrangements: independent living (*n* = 21), sheltered/supported accommodation (*n* = 6) Employment status: employed or had “regular daytime activity” (*n* = 14). SES and ethnicity not reported Setting: community	Participants youngest children were aged between 6 months and 18 years old 18 had 1–2 children and 9 had 3–6 children	Not stated	Expert by experience groups, providers of mental health services, and volunteers	“open-ended” interviews / TA (Miles & Huberman, 1994)	- Effects of MI on parenting—mothers - Effects of MI on parenting—fathers - Strategies for successful parenting
11.	Parrott et al. (2015), UK	To understand experiences of parents parenting roles maintained during admission to a secure forensic hospital	Of the total secure hospital population: schizophrenia (*n* = 100), PD (*n* = 7), Affective disorder (*n* = 4), unconfirmed (*n* = 4). Not possible to determine percentage of population by diagnosis	Mothers (*n* = 8) and fathers (*n* = 10) Living arrangements: medium secure hospital without children (*n* = 18) Other demographical information from the qualitative study was not provided Setting: inpatient	Fathers had 41 children between them (range = 1–5; median = 1). Mothers had 20 children between them (range = 1–3; (*M* = 2)	Not stated	Ward staff	Semi-structured interviews / Framework approach (Pope et al., 2000; Ritchie & Spencer, 1994)	- Parenthood and self-identity - Impact of MI on parenting - Parental concepts of offending and risk - Parenting from within the unit-maintaining relationships - Explaining MI and detention to children
12.	Rampou et al. (2015), South Africa	To explore and describe the parenting experiences of mothers with a chronic mental illness	Schizophrenia (*n* = 4; 40%), BD (*n* = 4; 40%), and MDD (*n* = 2; 20%)	Mothers (*N* = 10) Age (years): 40–49, (*n* = 6), 30–39 (*n* = 3), and 20–29 (*n* = 1) Relationship status: single, separated or widowed (*n* = 8), unknown (*n* = 2) Employment: employed (*n* = 1), unknown (*n* = 9). SES and ethnicity not reported Setting: community	Number and ages of children were not supplied	Not stated	Outpatient mental healthcare treatment and rehabilitation services	Individual interviews / Tesch’s descriptive method (Creswell, 2009)	- Challenges for mothers with regard to caring for their children - Family support needs
13.	Perera et al. (2014), Australia	To explore positive and challenging experiences of mothers with MI, from perspectives of mothers and HCPs	Primary diagnoses: schizophrenia (*n* = 5; 63%), MDD with psychotic symptoms (*n* = 2; 25%), BD (*n* = 1; 13%)	Mothers (*N* = 8) Relationship status: in relationship (*n* = 5), single (*n* = 3) Background: “Various cultural backgrounds including Polynesian and Indigenous Australian”. Ethnicity and SES not reported Setting: community	Mothers had a total of 20 children between them, aged 1–24 years 13 children were under the age of 10	Not stated	Adult public mental health service	Semi-structured interviews / GT (Charmaz, 2006)	- Positive aspects of motherhood for women living with MI - Challenging aspects of motherhood
14.	Tjoflåt and Ramvi (2013), Norway	To understand parenting with bipolar disorder	BD (*N* = 6; 100%)	Mothers (*n* = 5) and fathers (*n* = 1) Age (years): 31–50 (*M* = 41) Relationship status: married and shared parental responsibility (*n* = 3) divorced (*n* = 3) Employment: employed full- or part-time (*n* = 3), “national insurance” (*n* = 3). Living arrangements: renting (*n* = 3) own home (*n* = 3) Ethnicity and SES not reported Setting: community	Parents had 11 children between them, aged 1–18 years. Parents had between 1 and 3 children each	Not stated	Community mental health centres	Semi-structured interviews/IPA (Smith & Osborn, 2003)	- Balancing bipolar disorder and parenting - The need for support versus perceiving stigma - Dependence on their children - Change and growth
15.	Jungbauer et al. (2011), Germany	Explore the impact of parental schizophrenia on family members	Schizophrenia (*N* = 57; 100%)	Mothers (*n* = 40) and fathers (*n* = 17) Age (years): 19 – 54 (*M* = 38.3) Living arrangements: same household as child/ren (*n* = 36), separated from children (*n* = 18) Ethnicity and SES not reported Setting: inpatient and community	38 children took part aged 7 – 18 (*M* = 12; 19 males, 19 females)	ICD-10	Inpatient and outpatient psychiatric care facilities	Semi-structured interviews/GT and CA (Mayring, 2010)	- Everyday family life between crisis and normalisation - The perspective of sick parents - Effects on the couple relationship - Coping with stress and the consequences of stress in children - Family constellations
16.	Montgomery et al. (2011), Canada	To explore the experience of “hitting bottom” from the perspectives of mothers with SMI	Diagnoses were not detailed. The most common diagnosis was MDD	Mothers (*N* = 37) Age (years): 19–38 Living arrangements: lived with child/ren (*n* = 22), separated from child/ren (*n* = 10), unspecified (*n* = 5) Ethnicity and SES not reported Setting: inpatient and community	Children were aged 2–15	Not stated	N/A—secondary qualitative analysis	Semi-structured interviews/Narrative Analysis (Riessman, 2008)	- Storytellers - Stories of bottom
17.	Jungbauer et al. (2010), Germany	To investigate the experience of parenthood in parents with schizophrenia with young children and their needs for assistance	Schizophrenia (*n* = 17; 65%) and schizoaffective disorder (*n* = 9; 35%)	Mothers (*n* = 21) and fathers (*n* = 5) Mean age (years): 39.7 Living arrangements: lived with child/ren (*n* = 15), separated from children (*n* = 11), Employment: employed (*n* = 6), part-time (*n* = 5) Income: disability benefits (*n* = 13), unemployment benefit (*n* = 5), child-raising allowance (*n* = 1), no income (*n* = 1) Ethnicity not reported Setting: inpatient and community	Parents had an average of 1.8 children between the ages of 1 and 30 (*M* = 12.8)	ICD-10	Inpatient, semi-inpatient, and outpatient psychiatric facilities	Interview and oral survey/GT (Glaser, 1998) and CA (Mayring, 2010)	- Positive aspects/resources in the perception of parenthood - Negative aspects/burdens in the perception of parenthood - Support requests regarding parenting/upbringing
18.	Khalifeh et al. (2009), UK	To explore experiences, treatment preferences, and needs of mothers who were treated at home as an alternative to hospital admission for an acute severe mental health crisis	MDD (*n* = 10; 56%), BD (*n* = 6; 33%) and Schizophrenia (*n* = 2; 11%)	Mothers (*N* = 18) Age (years): 21–30 (*n* = 1), 31–40 (*n* = 9), 41–50 (*n* = 7), and 51–60 (*n* = 1) Ethnicity: White (*n* = 12), Black (*n* = 3) or Asian (*n* = 3) Living arrangements: alone (*n* = 9), with the child’s father (*n* = 8), with another male (*n* = 1) 5 children aged 12–18 also participated SES not reported Setting: community	Mothers had 1 (*n* = 6), 2 (*n* = 3), 3 (*n* = 5), or 4 (*n* = 4) children aged 0–1 (*n* = 3), 2–5 (*n* = 8), 6–11 (*n* = 13), 12–18 (*n* = 16), or > 18 (*n* = 3)	Not stated	Crisis resolution team	Semi-structured interviews/TA (Braun & Clarke, 2006)	- Mothers’ experiences: advantages and disadvantages - Child experiences
19.	Wilson and Crowe (2009), New Zealand	To explore how parents with bipolar disorder construct their role as parent, and how bipolar disorder is constructed in texts	BD (*N* = 6; 100%)	Mothers (*n* = 5), fathers (*n* = 1) No other socio-demographical details provided Ethnicity and SES not reported Setting: unclear	“Young children” (ages not reported) Number of children not reported	Self-identified diagnosis	Unclear	Semi-structured interviews/Critical discourse analysis (Titscher et al., 2000)	- Monitoring and Emotional Regulation
20.	Ueno and Kamibeppu (2008), Japan	To understand mothers’ perceptions of what experiences influence them or their parenting practices	Schizophrenia (*n* = 13; 65%) and mood disorders (*n* = 7; 35%)	Mothers (*N* = 20) Mean age (years) = 43 Relationship status: married (*n* = 14), separated or divorced (*n* = 4), widow (*n* = 1), never married (*n* = 1) Living arrangements: with children (*n* = 20) Ethnicity and SES not reported Setting: community	Mothers had between 1 and 3 children, ranging from 3 to 20 years old	DSM-IV-TR diagnosis	One psychiatric hospital and two psychiatric clinics	Semi-structured interviews/Modified GT (Kinoshita, 2003)	- Parenting whilst performing self-care - Balancing responsibilities - Feeling of affection for the child - Frustration with poor parenting - Feeling the child’s compassion
21.	Evenson et al. (2008), UK	To explore the experiences of fathers with psychosis	Schizophrenia (*n* = 7; 70%), schizoaffective disorder (*n* = 2; 20%), and delusional disorder (*n* = 1; 10%)	Fathers (*N* = 10) Age (years): 34–67 (*M* = 51) Relationship status: married (*n* = 5), cohabiting (N = 2), divorced (*n* = 1), single (*n* = 2) Ethnicity: “white” Living arrangements: with child/ren (*n* = 6), with partner/wife without child/ren (*n* = 2), alone (*n* = 2) SES not reported Setting: community	Participants had 21 children between them (7 girls, 14 boys aged 1–44 years)	Not stated	CMHTs	Semi-structured interviews/IPA (Smith & Osborn, 2003)	- Psychosis undermines the father–child relationship and the work of parenting - Pre-fatherhood aspirations - Fears for the children - Impact of psychosis on fathers
22.	Venkataraman and Ackerson (2008), USA	To understand the strengths, challenges, and service needs of mothers with BD	Bipolar-I disorder (*n* = 8; 80%) and Bipolar-II disorder (*n* = 2; 20%)	Mothers (*N* = 10) Age (years): 21–49 Relationship/living status: never married (*n* = 4), divorced (*n* = 1), widowed (*n* = 1), married/cohabiting (*n* = 4) Employment status: unemployed (*n* = 2), “lower level” jobs (*n* = 4), “higher level” jobs (*n* = 2), “a couple” were students SES: “low” (*n* = 7), “middle” (*n* = 3) Ethnicity: “white” (*n* = 10) Setting: community	The number of children in each family ranged from 1 to 4. Children were aged 1–30	Not stated	Community mental health centres and support groups	Semi-structured interviews/GT (Strauss, 1987)	- Strengths in Parenting - Challenges in Parenting - Service Needs
23.	Montgomery et al. (2006), Canada	To describe experiences of mothers with SMI and how they manage their mothering circumstance	Schizophrenia (*n* = 3; 15%), BD (*n* = 4; 20%), MDD (*n* = 9; 45%), and unspecified (*n* = 4; 20%)	Mothers (*N* = 20) Age (years): “early 20 s to late 30 s” All had contact with their children and 16 were living with their children Ethnicity and SES not reported Setting: inpatient and community	39 children between mothers, aged 2–15 years. Mothers had between one and four children	Self-identified	Referred by psychiatrists or “designate”	Unstructured formal interviews/GT (Glaser, 1998)	- Core category: appearing normal, creating security, being responsible - Keeping close: masking, censoring speech, doing mother work, seeking help
24.	Diaz-Caneja and Johnson (2004), UK	To understand the experiences of mothers with SMI and their views of the services they receive	Schizophrenia (*n* = 8; 36%) BD (*n* = 10; 45%) and severe depression with psychotic symptoms (*n* = 4; 18%)	Mothers (*N* = 22) Age (years): 20–29 (*n* = 2), 30–39 (*n* = 9), 40 + (*n* = 11) Ethnicity: White (*n* = 13), White European or South American (*n* = 3), Black UK (*n* = 1), Black Caribbean (*n* = 1), Black African (*n* = 1), Asian (*n* = 2), Mixed (*n* = 1) Relationship status: married/ cohabiting (*n* = 3), previously married and living alone (*n* = 11), widow (*n* = 1), never married (*n* = 7). Living arrangements: local authority (*n* = 21), privately renting (*n* = 1). Employment: part-time (*n* = 1), unspecified (*n* = 22). SES not reported Setting: community	Mothers had a total of 41 children 9 mothers had child/ren aged under 9 and 17 had child/ren aged 10–17 Child living arrangements: both parents (*n* = 2), mother only, (*n* = 9), father only (*n* = 4), other family member (*n* = 2), foster care (*n* = 4), adopted (*n* = 1)	Not stated	CMHT	Face-to-face semi-structured interviews/TA (Richards & Richards, 1998)	- Positive aspects of motherhood - Difficulties associated with motherhood - Effect of MI on children - Stigma - Views about services
25.	Savvidou et al. (2003), Greece	To explore the influence of the diagnosis of SMI on mother’s lives and relationships for mothers hospitalised on a psychiatric unit	Schizophrenia (*n* = 10; 50%), delusional disorder (*n* = 1; 5%), BD (*n* = 1; 5%), MDD (*n* = 3; 15%), and BPD (*n* = 2; 10%)	Mothers (*N* = 20) Age (years): 28–53 Living arrangements: hospitalised (*n* = 20); with child/ren (*n* = 13), without child/ren (*n* = 7) Relationship status: divorced (*n* = 10), unspecified (*n* = 10) Custody: lost custody (*n* = 10), regular contact (*n* = 1), full custody (*n* = 9) Ethnicity and SES not reported Setting: inpatient	Mothers had a total of 32 children between them, aged 3.5–18 years	DSM-IV diagnosis	Unclear	Face-to-face semi-structured interviews/Discourse Analysis (Burman & Parker, 1993; Parker, 1992)	- The discourse of “parenthood” - The discourses of “Mental Illness” and “Mentally Ill” parent - Relationship with partner, family, and social environment - MI and parenthood
26.	Ackerson (2003), USA	Explore how parents coped with the dual demands of parenthood and experiencing SMI	“Severe and persistent mental illness” (psychotic disorder or severe mood disorder); diagnostic information was not supplied	Mothers (*n* = 12), fathers (*n* = 1) Relationship status: married (*n* = 3), separated, widowed, or divorced (*n* = 10) Ethnicity and SES not reported Setting: community	Children’s ages were not stated	Self-identified or ‘documented’ diagnosis	Community mental health centres or mental health consumer groups	1:1 semi-structured / structured interviews/Guided by GT (Glaser & Strauss, 1967)	- Problems with diagnosis and treatment - Stigma and discrimination - Chaotic interpersonal relationships - The strain of single parenthood - Custody issues - Relationship with children: discipline, boundary issues, role reversal - Social support - Pride in being a parent
27.	Thomas and Kalucy(2002), Australia	To explore the views of parents and their families about the impact of mental illness on their families, especially their children	BD (*n* = 11; 31%), MDD (*n* = 10; 29%), schizoaffective disorder (*n* = 8; 23%), schizophrenia (*n* = 3; 9%), PD (*n* = 2; 6%), or PND (*n* = 1; 3%)	Mothers (*n* = 28) and fathers (*n* = 7) Age (years): 30–67 (*M* = 44.6) Living arrangements: separated from child/ren completely (*n* = 12), lived with some of their child/ren (*n* = 4) Ethnicity and SES not reported Setting: inpatient and community	Parents had 88 children between them (48 daughters and 40 sons) aged 2–36 years	Not stated	Inpatient and outpatient mental health services and consumer groups	Semi-structured interviews/Unspecified qualitative methodology	- Impact on daily life - Family concerns - Hospitalisation - Ongoing management - Participant recommendations
28.	Nicholson et al. (1998), USA	Understand the parenting experiences of women with mental illness from the perspectives of mothers and case managers	Affective disorder (*n* = 23; 55%), psychotic disorder (*n* = 8; 19%), anxiety disorder (*n* = 6; 14%), or “other/don’t know” (*n* = 5; 12%)	Mothers (*N* = 42) Age (years): 22–48 (*M* = 35) Relationship status: married/ cohabiting (*n* = 19), previously married (*n* = 20), never married (*n* = 3) Living arrangements: private home (*n* = 34), group care (*n* = 4), family home (*n* = 3), hospital (*n* = 1) Ethnicity: Caucasian (*n* = 35), African American (*n* = 5), Hispanic/Latina (*n* = 1), Native American (*n* = 1) Setting: community	97 children between mothers with an average of 2.2 children per family Living: with mother/ mother and partner (*n* = 38), with father (*n* = 18), foster care (*n* = 6), adopted (*n* = 10), with relatives (*n* = 10), independently (*n* = 5)	Not stated	Case management services/ Unspecified thematic analysis	Face-to-face focus groups / Unspecified TA	- The stigma of MI - Day-to-day parenting - Managing MI - Custody of and contact with children
29.	Sands (1995), USA	To examine mothers’ perceptions of themselves as mothers and persons with mental illness, their psychosocial issues, and their receptivity to support programmes	Schizophrenia (*n* = 6; 60%), schizotypal personality disorder (*n* = 1; 10%), MDD (*n* = 1; 10%), BD (*n* = 1; 10%) and unknown (*n* = 1; 10%)	Mothers (*N* = 10) Age (years): 21–37 (*M* = 27) SES: low-income (*n* = 10) Living arrangements: community supervised apartment (*n* = 10). Mothers’ children were living with them (*n* = 5) or in foster care or with a relative (*n* = 5) Ethnicity: African American (*n* = 7) or White (*n* = 3) 8 mothers without SMI were included as a comparison group Setting: community	Ages and number of children not specified	Not stated	Support services	Informal conversations and semi-structured interviews/Unspecified TA	- Experience of motherhood and MI - Programme demands - Psychosocial issues

^a^Other methods of data collection may have been used but only data gathered from interviews or focus groups are included in the review

*SES* socio-economic status, *BD* bipolar disorder, *MDD* major depressive disorder, *PND* post-natal depression, *BPD* borderline personality disorder, *PTSD* post-traumatic stress disorder, *OCD* obsessive compulsive disorder, *PD* personality disorder, *ADHD* attention deficit hyperactivity disorder, *CMHT* community mental health team, *TA* thematic analysis, *GT* grounded theory, *CA* content analysis, *IPA* interpretive phenomenological analysis; *MI* mental illness

### Methodological Quality of Included Studies

Overall, the methodological quality of the 29 studies was assessed as being high (*n* = 13) or moderately high (*n* = 16; see Table 3). However, only one study adequately considered the researcher–participant relationship (Chan et al., 2019), 16 studies (55.17%) adequately took ethical issues into consideration, and five studies (17.24%) demonstrated an absence of ethical considerations. Chan et al. (2019), Sabella et al. (2022), Chen et al., (2021b), and Montgomery et al. (2006) received the highest methodological quality ratings of 9.5/10 or above, whereas Sands (1995), Thomas and Kalucy (2002), and Venkataraman and Ackerson (2008) received the lowest quality ratings of 7/10 or lower. As no widely accepted approach for excluding qualitative studies on the basis of quality exists (Dixon-Woods et al., 2006; Thomas & Harden, 2008), no studies were excluded from this review on the grounds of quality.

**Table 3:** Methodological quality assessment of included studies

	Study: Authors and year	Was there a clear statement of the aims of the research	Is a qualitative methodology appropriate	Was the research design appropriate to address the aims of the research	Was the recruitment strategy appropriate to the aims of the research	Was the data collected in a way that addressed the research issue	Has the relationship between researcher and participants been adequately considered	Have ethical issues been taken into consideration	Was the data analysis sufficiently rigorous	Is there a clear statement of findings	How valuable is the research	Quality Appraisal (total score)
1	Radley et al. (2022a)	Yes (1)	Yes (1)	Yes (1)	Yes (1)	Yes (1)	PA (0.5)	Yes (1)	Yes (1)	Yes (1)	Yes (1)	High (9.5)
2	Sabella et al. (2022)	Yes (1)	Yes (1)	Yes (1)	Yes (1)	Yes (1)	No (0)	Yes (1)	Yes (1)	Yes (1)	Yes (1)	High (9.5)
3	Chen et al. (2021b)	Yes (1)	Yes (1)	Yes (1)	Yes (1)	Yes (1)	PA (0.5)	Yes (1)	Yes (1)	Yes (1)	Yes (1)	High (9.5)
4	Mulvey et al. (2021)	Yes (1)	Yes (1)	PA (0.5)	Yes (1)	Yes (1)	No (0)	PA (0.5)	PA (0.5)	Yes (1)	Yes (1)	Moderate (7.5)
5	Boström and Strand (2021)	Yes (1)	Yes (1)	Yes (1)	Yes (1)	Yes (1)	No (0)	Yes (1)	Yes (1)	Yes (1)	Yes (1)	High (9)
6	Strand et al. (2020)	Yes (1)	Yes (1)	PA (0.5)	Yes (1)	Yes (1)	PA (0.5)	Yes (1)	Yes (1)	Yes (1)	Yes (1)	High (9)
7	Chan et al. (2019)	Yes (1)	Yes (1)	Yes (1)	Yes (1)	Yes (1)	Yes (1)	Yes (1)	Yes (1)	Yes (1)	Yes (1)	High (10)
8	Awram et al. (2017)	Yes (1)	Yes (1)	Yes (1)	Yes (1)	Yes (1)	No (0)	Yes (1)	Yes (1)	Yes (1)	Yes (1)	High (9)
9	Klausen et al. (2016)	Yes (1)	Yes (1)	Yes (1)	PA (0.5)	PA (0.5)	No (0)	PA (0.5)	Yes (1)	Yes (1)	Yes (1)	Moderate (7.5)
10	van der Ende et al. (2016)	Yes (1)	Yes (1)	Yes (1)	Yes (1)	Yes (1)	No (0)	PA (0.5)	Yes (1)	Yes (1)	Yes (1)	Moderate (8.5)
11	Parrott et al. (2015)	Yes (1)	Yes (1)	Yes (1)	Yes (1)	Yes (1)	No (0)	Yes (1)	Yes (1)	Yes (1)	Yes (1)	High (9)
12	Rampou et al. (2015)	Yes (1)	Yes (1)	Yes (1)	Yes (1)	Yes (1)	No (0)	Yes (1)	Yes (1)	Yes (1)	Yes (1)	High (9)
13	Perera et al. (2014)	Yes (1)	Yes (1)	Yes (1)	Yes (1)	Yes (1)	No (0)	PA (0.5)	Yes (1)	Yes (1)	Yes (1)	Moderate (8.5)
14	Tjoflåt and Ramvi (2013)	Yes (1)	Yes (1)	Yes (1)	Yes (1)	Yes (1)	No (0)	Yes (1)	Yes (1)	Yes (1)	Yes (1)	High (9)
15	Jungbauer et al. (2011)	Yes (1)	Yes (1)	Yes (1)	Yes (1)	Yes (1)	No (0)	Yes (1)	Yes (1)	Yes (1)	Yes (1)	High (9)
16	Montgomery et al. (2011)	Yes (1)	Yes (1)	Yes (1)	Yes (1)	PA (0.5)	No (0)	PA (0.5)	Yes (1)	Yes (1)	Yes (1)	Moderate (8)
17	Jungbauer et al. (2010)	Yes (1)	Yes (1)	Yes (1)	Yes (1)	Yes (1)	No (0)	No (0)	Yes (1)	Yes (1)	Yes (1)	Moderate (8)
18	Khalifeh et al. (2009)	Yes (1)	Yes (1)	PA (0.5)	Yes (1)	Yes (1)	No (0)	Yes (1)	Yes (1)	Yes (1)	Yes (1)	Moderate (8.5)
19	Wilson and Crowe (2009)	Yes (1)	Yes (1)	Yes (1)	PA (0.5)	PA (0.5)	No (0)	Yes (1)	Yes (1)	Yes (1)	Yes (1)	Moderate (8)
20	Ueno and Kamibeppu (2008)	Yes (1)	Yes (1)	PA (0.5)	Yes (1)	Yes (1)	No (0)	Yes (1)	Yes (1)	Yes (1)	Yes (1)	Moderate (8.5)
21	Evenson et al. (2008)	PA (0.5)	Yes (1)	Yes (1)	Yes (1)	Yes (1)	PA (0.5)	PA (0.5)	Yes (1)	Yes (1)	Yes (1)	Moderate (8)
22	Venkataraman and Ackerson (2008)	Yes (1)	Yes (1)	PA (0.5)	PA (0.5)	Yes (1)	PA (0.5)	No (0)	PA (0.5)	Yes (1)	Yes (1)	Moderate (7)
23	Montgomery et al. (2006)	Yes (1)	Yes (1)	Yes (1)	Yes (1)	Yes (1)	PA (0.5)	Yes (1)	Yes (1)	Yes (1)	Yes (1)	High (9.5)
24	Diaz-Caneja and Johnson (2004)	Yes (1)	Yes (1)	Yes (1)	Yes (1)	Yes (1)	No (0)	Yes (1)	Yes (1)	Yes (1)	Yes (1)	High (9)
25	Savvidou et al. (2003)	Yes (1)	Yes (1)	Yes (1)	Yes (1)	Yes (1)	No (0)	PA (0.5)	PA (0.5)	Yes (1)	Yes (1)	Moderate (8)
26	Ackerson (2003)	Yes (1)	Yes (1)	Yes (1)	PA (0.5)	Yes (1)	No (0)	No (0)	Yes (1)	Yes (1)	Yes (1)	Moderate (7.5)
27	Thomas and Kalucy (2002)	Yes (1)	Yes (1)	PA (0.5)	PA (0.5)	Yes (1)	No (0)	No (0)	PA (0.5)	Yes (1)	Yes (1)	Moderate (6.5)
28	Nicholson et al. (1998)	Yes (1)	Yes (1)	Yes (1)	Yes (1)	Yes (1)	No (0)	PA (0.5)	PA (0.5)	Yes (1)	Yes (1)	Moderate (8)
29	Sands (1995)	Yes (1)	Yes (1)	Yes (1)	PA (0.5)	PA (0.5)	No (0)	No (0)	PA (0.5)	Yes (1)	PA (0.5)	Moderate (6)
	Percentage of studies rated ‘Yes’ (1)	96.6%	100%	75%	79.3%	86.2%	3.4%	55.2%	79.3%	100%	96.6%	

*PA* Partially agree

### Thematic Synthesis

**Fig. 2:**
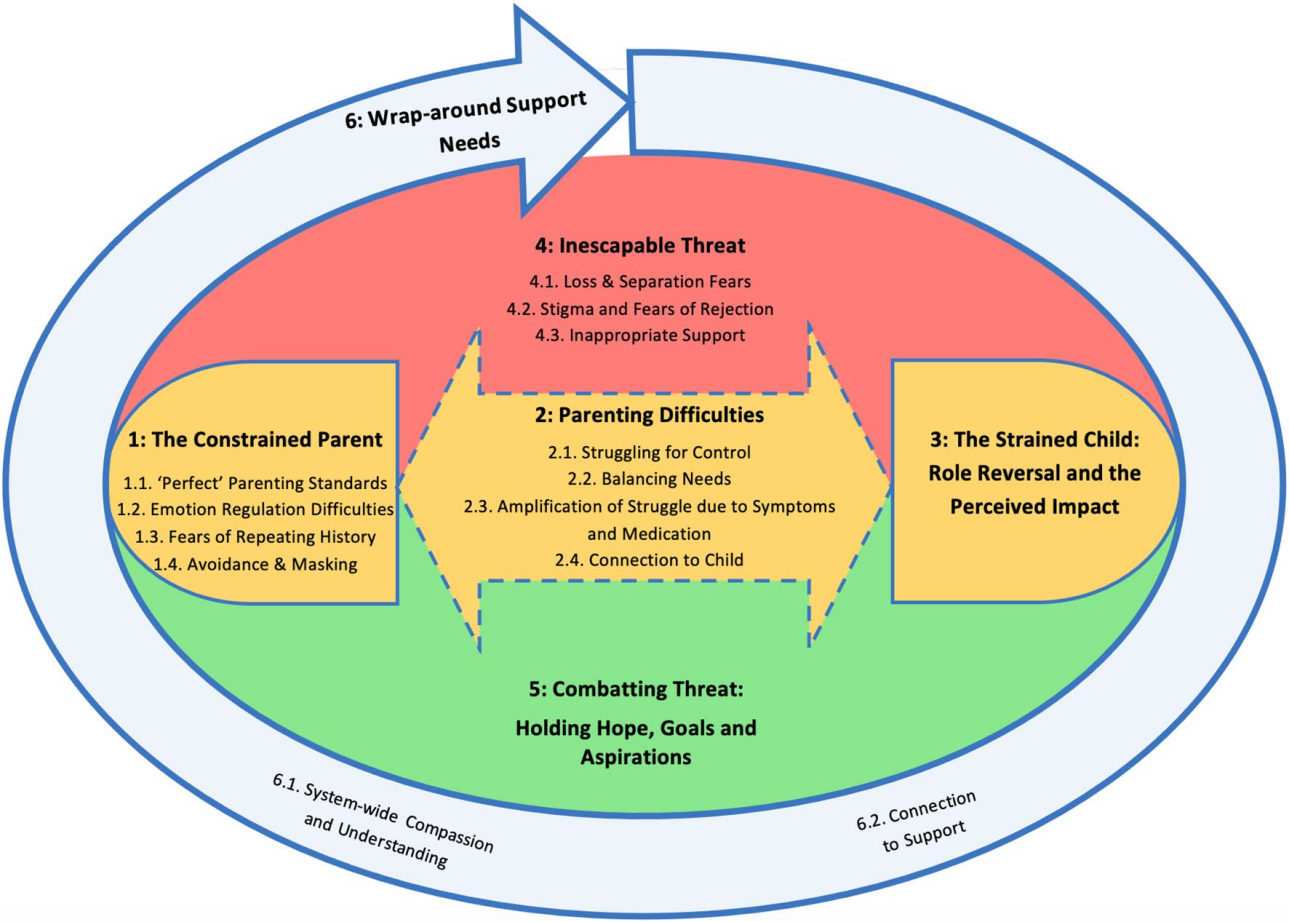
Conceptual model depicting themes and sub-themes

Six themes were conceptualised to represent how parenting is influenced by experiences of SMI: (1) The Constrained Parent, (2) Parenting Difficulties, (3) The Strained Child, (4) Inescapable Threat, (5) Combatting Threat and (6) Wrap-around Support Needs. A conceptual model was developed (Fig. 2), illustrating the relationship between the six main themes and 13 sub-themes. The model depicts the centrality of SMI-related parenting difficulties in generating strain on parent–child relationships, the all-consuming and invasive role of threat on parenting, and the strategies that parents used to combat such difficulties. The need for comprehensive and inclusive system-wide support is indicated. Illustrative quotes are provided within the text in italics (Supplementary Material 2 presents additional exemplar quotes). A matrix of themes (Table 4) illustrates which themes were present in the included studies.

**Table 4:** Matrix of theme representation within the included studies

	Study: Authors and year	Theme 1: The constrained Parent				Theme 2: Parenting Difficulties				Theme 3: The Strained Child: Role Reversal and the Perceived Impact	Theme 4: Inescapable Threat			Theme 5: Combatting Threat: Holding Hope, Goals and Aspirations	Theme 6: Wrap-around Support Needs	
		Perfect Parenting Standards	Emotion Regulation Difficulties	Fears of Repeating History	Avoidance and Masking	Struggling for Control	Balancing Needs	Amplification of Struggle due to Symptoms and Medication	Connection to Child		Loss and Separation Fears	Stigma and Fears of Rejection	Inappropriate Support		System-wide Compassion and Understanding	Connection to support
1	Radley et al. (2022a )	✓	–	✓	✓	✓	✓	✓	✓	✓	✓	✓	–	✓	✓	✓
2	Sabella et al. (2022)	✓	✓	✓	–	✓	✓	✓	✓	✓	✓	✓	✓	✓	–	–
3	Chen et al. ( 2021b)	✓	✓	✓	✓	✓	✓	✓	✓	✓	–	✓	✓	✓	✓	✓
4	Mulvey et al. (2021)	✓	✓	✓	✓	✓	✓	✓	✓	✓	✓	✓	✓	✓	✓	✓
5	Boström and Strand (2021)	✓	✓	✓	✓	✓	–	✓	✓	✓	–	–	–	✓	–	✓
6	Strand et al. (2020)	✓	✓	✓	✓	✓	✓	✓	✓	✓	✓	✓	✓	✓	✓	✓
7	Chan et al. (2019)	✓	✓	✓	✓	✓	✓	✓	✓	✓	–	✓	✓	–	✓	✓
8	Awram et al. (2017)	✓	–	–	✓	–	✓	✓	✓	✓	✓	✓	–	✓	✓	✓
9	Klausen et al. (2016)	✓	✓	✓	✓	✓	✓	–	✓	✓	✓	✓	✓	–	✓	✓
10	van der Ende et al. (2016)	✓	✓	✓	✓	✓	✓	✓	✓	–	–	✓	✓	✓	✓	✓
11	Parrott et al. (2015)	✓	✓	–	✓	✓	✓	✓	✓	–	✓	✓	✓	✓	✓	✓
12	Rampou et al. (2015)	✓	✓	–	–	✓	✓	✓	✓	✓	✓	✓	✓	–	✓	✓
13	Perera et al. (2014)	✓	✓	✓	✓	✓	✓	✓	✓	✓	✓	✓	–	✓	✓	✓
14	Tjoflåt and Ramvi (2013)	✓	✓	✓	–	✓	✓	✓	✓	✓	–	✓	✓	✓	✓	✓
15	Jungbauer et al. (2011)	✓	–	✓	✓	✓	✓	✓	✓	✓	✓	✓	–	–	✓	✓
16	Montgomery et al. (2011)	✓	✓	–	✓	✓	✓	✓	✓	✓	✓	–	✓	–	✓	✓
17	Jungbauer et al. (2010)	✓	–	✓	✓	✓	✓	✓	✓	✓	✓	✓	✓	✓	✓	✓
18	Khalifeh et al. (2009)	✓	✓	–	–	✓	✓	✓	✓	✓	✓	✓	✓	–	✓	✓
19	Wilson and Crowe (2009)	✓	✓	–	✓	–	✓	✓	–	–	–	✓	✓	–	–	–
20	Ueno and Kamibeppu (2008)	–	✓	–	✓	–	✓	✓	✓	✓	–	✓	–	✓	–	–
21	Evenson et al. (2008)	✓	–	✓	✓	✓	✓	✓	✓	✓	✓	✓	✓	✓	–	–
22	Venkataraman and Ackerson (2008)	–	✓	✓	✓	✓	✓	✓	✓	✓	✓	–	✓	✓	✓	✓
23	Montgomery et al. (2006)	✓	✓	–	✓	✓	✓	✓	✓	–	✓	–	✓	✓	✓	✓
24	Diaz-Caneja and Johnson (2004)	✓	–	✓	✓	✓	✓	✓	✓	✓	✓	✓	✓	✓	✓	✓
25	Savvidou et al. (2003)	✓	✓	–	✓	✓	✓	✓	✓	✓	✓	✓	✓	✓	✓	✓
26	Ackerson (2003)	✓	✓	–	–	✓	✓	✓	✓	✓	✓	✓	✓	✓	✓	✓
27	Thomas and Kalucy (2002)	–	✓	–	✓	✓	✓	✓	✓	✓	✓	–	✓	–	✓	✓
28	Nicholson et al. (1998)	✓	✓	✓	–	✓	✓	✓	✓	–	✓	✓	✓	–	–	–
29	Sands (1995)	✓	✓	–	–	✓	✓	✓	✓	✓	✓	✓	✓	✓	✓	✓

✓  Theme identified; – Theme not identified

### Theme 1: The Constrained Parent

Feeling bound by the impact of experiences of SMI was a common theme across studies. Parents perceived the “*overwhelming*” (Mulvey et al., 2021, p. 18) nature of SMI to exacerbate the “*pressure*” (Perera et al., 2014, p. 174) associated with being a parent: “*…I couldn’t be a parent… I couldn’t be a mum. I wasn’t capable. I literally was not capable of being a parent because I was so ill” (*Radley et al., 2022a* p.5).* There was often a sense that parents felt hopeless and frustrated; feelings that compelled parents to adopt “*self-restrained*” (Chen et al., 2021b, p. 6) parenting. This constrained parenting style was conceptualised to be a protective defence, consequent of parental comparisons to idealised parenting standards, difficulties regulating emotions, and worries about negatively impacting children. This theme consisted of four sub-themes.

### Subtheme 1.1: “Perfect” Parenting Standards

Parents appeared bound by a “*tremendous guilt*” (Montgomery et al., 2011, p. 4) about their identity of being a parent who experienced mental health difficulties. Societal ideas about “*perfect*” and “*ideal*” parenting (Chen et al., 2021b, p. 5) were conceptualised as unattainable standards that served to perpetuate parental perceptions of inadequacy and incompetence. A sense of threat and vulnerability associated with such perceptions existed for many. By ‘hiding’ themselves during periods of significant distress, including from their children, parents attempted to protect their valued parenting identities. However, distance in the parent–child relationship could be an unintended consequence, serving to further perpetuate parental perceptions of failure: “*I fail both as a person and mother”* (Chan et al., 2019, p. 532). Consequently, parents would “*second guess*” their capacity to be “*good*” parents (Perera et al., 2014, p. 177), which conflicted with parental instincts to protect and be close to their children.

*“When I was psychotic, I stayed away for long periods. I didn’t want her [child] to see me in such bad shape*” (Strand et al., 2020, p. 623).

### Subtheme 1.2: Emotion Regulation Difficulties

Parents frequently struggled to manage difficult feelings associated with “*stressful*” (Sabella et al., 2022, p. 6) and “*scary*” parenting circumstances (Strand et al., 2020, p. 628), which in many cases perpetuated isolation and disconnection. Difficult emotions and circumstances were regarded as inescapable for some, leading to feelings of being trapped and reflecting “*helplessness*” (Chen et al., 2021b, p. 5). Such powerful feelings were often internalised: “*You have so much pain you do not know where it goes so you turn it inward on yourself*” (Montgomery et al., 2011, p. 5). An overwhelming desire to escape this pain was frequently reported. For some, avoidance and substance use provided temporary relief, whilst others perceived suicide to be their only option.

*“When my first son was 1 year old, I was suicidal. I felt bad as a parent. I could not fulfil the mother role”* (van der Ende et al., 2016, p. 90).

Other parents demonstrated an externalisation of uncontainable emotions. This was often associated with a limited window of tolerance during which parents reported getting “*angry very easily*” with their children (Venkataraman & Ackerson, 2008, p. 398). Often, parents appeared to struggle with managing difficult emotions and situations effectively. This could sometimes result in excessive child discipline, further distancing parents from their children: “*I couldn’t control myself. I couldn’t even after I hit her*” (Chan et al., 2019, p. 533).

### Subtheme 1.3: Fears of Repeating History

Pervasive parental fears about passing on difficult mental health experiences to their children were common and could be conceptualised as an unwelcome family legacy: “*I feel as it goes from son to son this thing you know?*” (Evenson et al., 2008, p. 636). Parenting style was shaped profoundly by such fears, whilst a sustained impact of parents’ own experience of being parented was also evident. For some, a lenient parenting style was adopted, driven by fears of exposing children to painful emotions related to neglectful or abusive parenting they had themselves suffered, particularly when painful memories involving shame reactions were triggered by interactions with their children. Several authors (i.e. Ackerson, 2003; Montgomery et al., 2006; Tjoflåt & Ramvi, 2013; Venkataraman & Ackerson, 2008) suggested that parents’ own apparent insecure attachment representation led some to seek an especially close bond with their children and many parents wished to protect their children from the adverse childhood experiences they had endured themselves. Across studies, parents valued secure, safe, and consistent care and this was particularly important for parents who did not have secure early attachment relationships. When this was absent in their childhood, providing this for their children was considered a priority.

*“…It [childhood home] was just not a safe place…so for [daughter], I have tried to provide her with like a really safe place to be that is clean and I am always there”* (Venkataraman & Ackerson, 2008, p. 395).

### Subtheme 1.4: Avoidance and Masking

Attempting to remedy stigma, fear, and shame, parents described using a “*shield*” (Jungbauer et al., 2010, p. 236) and “*tried to hide*” their authentic selves (Tjoflåt & Ramvi, 2013, p. 87), creating an illusion of ‘perfect parenting’ to satisfy the expectations held by themselves, their children and society to “*pretend that things were OK*” (Montgomery et al., 2011, p. 4). In the presence of perceived power figures, parents could become exhausted by masking their difficulties and “*trying to entertain everyone in the room*” (Parrott et al., 2015, p. 266), particularly when a pressure to demonstrate parenting capacity to child protection services was experienced.

Avoidance and withdrawal were strategies enlisted when parents described feeling overwhelmed. Some found such strategies helpful, offering themselves time to self-regulate and subsequently return to parenting: *“I would walk away, take a toilet break, or drink a cup of water. Then I would deal with our emotions later*” (Chan et al., 2019, p. 532). However, some parents recognised such strategies were only temporarily effective and emotions remained unprocessed and burdensome. In the face of difficult symptoms, some parents learnt specific strategies to manage difficult symptoms, whilst others adapted a “*mechanical*” parenting mode (Montgomery et al., 2006, p. 24) to persevere parental functioning:

*“…I was depressed enough so that I just kind of went through life. I didn’t feel anything, I just, you know, did the grocery shopping, did the cooking, took care of their needs, but I wasn’t happy…”* (Venkataraman & Ackerson, 2008, p. 397).

### Theme 2: Parenting Difficulties

The impact of parenting difficulties on the positioning of parent and child roles, which were often polarised, was conceptualised within this theme*.* Parenting difficulties were impacted by specific SMI-related factors, including symptom and medication effects, alongside other factors including connection, understanding, and parent–child bonding. This theme consisted of four sub-themes.

### Subtheme 2.1: Struggling for Control

Mothers and fathers who parented at home as well as from inpatient settings struggled with asserting boundaries, maintaining discipline, and managing routines. Exhaustion and fatigue were frequently referenced, and parents often reported feeling depleted of the energy required to assert boundaries: “*The children walked over me; I could not keep standing because of the burden of my depression*” (van der Ende et al., 2016, p. 91). In response, parents described often withdrawing from interacting with their children. However, parental awareness of their children’s needs could result in cycles of guilt and resentment between parents and their children. Some parents managed by displacing responsibilities onto their children or by directly communicating their vulnerability. However, when communication difficulties existed, some parents recognised that they used excessive discipline to re-gain control. Conversely, some parents avoided asserting boundaries entirely, describing themselves as being “*too kind*” (Boström & Strand, 2021, p. 72) which could lead to blurred parent and child roles. In such cases, parents’ desire to be unconditionally loved and accepted appeared to inhibit their ability to assert boundaries: *“I think sometimes I am more of a friend and I think that’s my downfall…”* (Venkataraman & Ackerson, 2008, p. 400).

### Subtheme 2.2: Balancing Needs

Parents were significantly challenged by the competing demands of parenting whilst experiencing SMI. Parents recognised the dilemma of balancing their own needs for respite with their children’s needs for attention, comfort, and connection: “*What comes first? Me sleeping or me being available for my child?*” (Perera et al., 2014, p. 176). The energy required to sustain adequate balancing of demands was easily depleted. Trapped in an unsustainable tug of war, parents experienced profound guilt and perceptions of inadequacy when defeated by exhaustion.

*“I try to keep my balance, for when I am terribly tired and feel bad, I push myself as much as I can, and I feel bad, it hurts not to have enough strength for my children…”* (Tjoflåt & Ramvi, 2013, p. 82).

Although parents largely recognised the importance of meeting their own needs to be able to meet the needs of their children, their ability to effectively balance was thwarted by the confines of busy family lives. Consequently, parents’ own needs were often neglected. *“…I couldn’t run the whole struggle, not even look after myself, much less to look after a child*” (Khalifeh et al., 2009, p. 637).

### Subtheme 2.3: Amplification of the Struggle due to Symptoms and Medication

Parental mental health difficulties were associated with an amplified parenting “*struggle*” (Evenson et al., 2008, p. 637) that some conceptualised as “*a living hell*” (Montgomery et al., 2006, p. 23). Parenting ability could be negatively impacted by cognitive difficulties, particularly during periods of significant psychological distress *“Sometimes I would forget to bath them for 4 or 5 days*” (Thomas & Kalucy, 2002, p. 42). Fear, shame, and guilt appeared to be felt profoundly when parents did not understand why their children were incorporated into their symptoms, particularly when thoughts of harming their children conflicted with their instincts to protect.

*“…regardless of how I loved my [child] I had thoughts of hurting her, so I have to put her down and I couldn’t understand why I had these thoughts”* (Montgomery et al., 2006, p. 24).

Emotional and physical closeness within parent–child dyads appeared to be influenced by parental reactions to these threatening experiences. Some parents responded by seeking closeness to their children due to fears of custody loss or other harm coming to their children, whilst others distanced themselves to protect their children from their thoughts. When children themselves were perceived as being the threat, harm to children could arise.

*“…I was hallucinating that there was demons inside of him so I took a knife sharpener and just pressed it on his chest…So I didn’t really attack him, in my mind I was protecting myself”* (Mulvey et al., 2021, p. 14).

A widely recognised parenting difficulty involved fatigue and low motivation which often impacted parent–child interactions. Medication side effects were frequently reported to amplify exhaustion, which could compound parenting difficulties. For some, medication was conceptualised as a “*mental straitjacket*” (Evenson et al., 2008, p. 635), further constraining parents’ sense of control. However, symptom effects were positively conceptualised when parents had increased energy, for example, during manic episodes. In such circumstances, parents benefited from energy related to their experience of mania that had been previously depleted, whilst children benefited from parents who were more physically present.

### Subtheme 2.4: Connection to Child

Parents’ desire to be “*close*” (Montgomery et al., 2006, p. 23) to their children was often thwarted by parental feelings of being overwhelmed and “*consumed*” (Perera et al., 2014, p. 175) by their mental health difficulties. Consequently, parents often appeared unable to co-regulate and emotionally connect with their children: “*It’s very difficult when you’re wrapped up in your own emotional needs to look at the emotional needs that your children have*” (Diaz-Caneja & Johnson, 2004, p. 476). For some parents, their ability to feel connected with their children was compounded by an absence of emotional connection, which could result in perceptions of polarised and emotionally distanced parent–child relationships:

*“…it is as if we are somehow not together; you know, it is as if I am in my own world, pondering on things and then the children wonder why you are so distant”* (Tjoflåt & Ramvi, 2013, p. 82).

Other studies reported parental difficulties in distinguishing their child’s emotions from their own. A ‘special bond’, within which children could be conceptualised as parents’ “*soul mates*” (Ackerson, 2003, p. 115) was experienced by some and appeared to represent parents’ desire to attain unconditional acceptance. Whether parents were “*insightful*” (Parrott et al., 2015, p. 265) about their own and their children’s needs played a central role in parental approaches to communicating with their children about mental health difficulties. When knowledge was perceived to be lacking, avoidance of discussions was often reported:

“*They don’t really understand my illness, and I don’t understand my illness either, so it’s so hard to talk about it...”* (Khalifeh et al., 2009, p. 637).

Others avoided discussions due to shame, fear of damaging their child, or believing that discussions were unnecessary, which could create a communication barrier between parent and child dyads. However, other parents reported it was important for children to be informed about their mental health difficulties and foster age-appropriate conversations with their children that were “*less scary*” (Awram et al., 2017, p. 154).

### Theme 3: The Strained Child

Parents often relied on their children to meet their needs and all studies reflected that family relationships were strained. The “*chaos*” (Montgomery et al., 2006, p. 23) of parenting was frequently displaced onto children, who parents reported could become strained with the heavy demands placed upon them to satisfy roles that were often incongruous with their developmental age. When parents conceptualised themselves as vulnerable and child-like, parents reflected that their children often sacrificed their own needs to care for them. Parental shame and guilt were felt profoundly when the impact on children was realised, particularly where parent–child role reversals were experienced:

*“I’m reliant on him physically to go to bed, physically to get up, emotionally because he’s my one and only contact. And it’s almost like sometimes I am the child, and he’s the parent”* (Khalifeh et al., 2009, p. 636).

Reflective of heightened threat perceptions, parents perceived their children to be fearful of harm coming to family relationships and observed their children to adopt strategies intended to protect these relationships by assuming parenting roles. Parent–child role confusion was felt profoundly when parents attempted to transition back into previously established parenting positions following acute episodes of psychological distress. For example, following hospital admissions during which parents and their children lived separately, the restatement of boundaries and control was particularly difficult to navigate.

*“…my daughter got herself a little job, she left school ... she was like running the show, being the mum, and I was just like a puppet”* (Perera et al., 2014, p. 175).

Parental guilt and shame were heavily cited across studies in relation to parents’ worry about the impact of their mental health difficulties on their children’s social, emotional, and academic development. Although the voices of children were not included in the current review, some parents described believing that their expressions of distress placed unfair strain on their children, with difficulties “*invading their lives*” (Montgomery et al., 2011, p. 4). These parents observed their children to demonstrate particular concern and responsibility for relieving their distress. However, children’s responses to parental distress varied across families. Some parents perceived their children to be “*sick and tired*” (Thomas & Kalucy, 2002, p. 45) of the unpredictability of their mental health difficulties and some observed that their emotional and physical absences could leave their children feeling isolated and alone:

“*She [daughter] felt like she was living on an island. She missed the support she needed from me, during my depression*” (van der Ende et al., 2016, p. 90).

Some parents described profound emotional distress to be experienced by their children, a possible manifestation of parents lacking knowledge about how to support their children emotionally. Some parents conceptualised child behavioural difficulties to be deliberate attempts to exacerbate their own stress, rather than their child’s attempt to communicate their own distress. This lack of parental understanding and possible co-dysregulation could serve to further isolate children and their emotional needs.

*“…Because he has been depressed, down in the dumps. He got hold of knife two weeks ago and had it close to his wrist and ready to cut himself and I asked him why he did it ‘I don’t know mommy’”* (Venkataraman & Ackerson, 2008, p. 402).

### Theme 4: Inescapable Threat

A relentless and inescapable power of threat permeated across multiple areas of parents’ lives. Parenting difficulties appeared to be amplified by constant and dominating fears of child loss, negative self-perceptions that threatened parents’ sense of parenting competence, inescapable societal stigma that threatened parents’ sense of acceptance and safety in the communities they lived within, and overwhelming feelings of being inappropriately supported by the systems they hoped would support them. The role of inescapable threat appeared to cause parents to become increasingly consumed by fear and less able to seek support, further perpetuating feelings of being constrained and bound by parenting difficulties. Three sub-themes were established.

### Subtheme 4.1: Loss and Separation Fears

A fundamental and widespread barrier to parents talking about their mental health difficulties and seeking support were profound fears of custody loss. This fear existed both for parents who had previously experienced child removal and those who feared it: “*Every mother’s fear is that her children will be taken into care*” (Diaz-Caneja & Johnson, 2004, p. 477). Parents experienced contact with child protection agencies as “*traumatic*” and “*intimidating*” (Perera et al., 2014, p. 177), fearing the consequences of being negatively evaluated. Parents frequently avoided services and hid their authentic selves, attempting protect custody of their children.

“*I didn’t want to go to a psychiatrist because I thought he would lock me up and I wanted to raise my kids*” (Ackerson, 2003, p. 112).

When separations did occur, parents reported feeling imprisoned and isolated, consumed by sadness and shame: “*My heart is in chains. It never gets easy, not for any mother; that pain never completely goes away*” (Nicholson et al., 1998, p. 639). Whilst separations threatened parent–child relationships, parents largely remained committed to contact with their children, demonstrating their need to remain emotionally and physically connected. Some parents recognised when custody arrangements were in their children’s best interests; however, parents commonly reported experiencing shame and humiliation during the process of attempting to re-gain child contact. Together, these experiences served to act as powerful barriers to parents accessing services and talking about their needs, serving to further isolate parents and children from accessing support.

### Subtheme 4.2: Stigma and Fears of Rejection

Integral to parenting capacity was how safe and secure parents felt, both within themselves, their family systems, and wider society. A significant barrier to safety was societal stigma, where dominant discourses about parents with mental health difficulties being “*dangerous”* (Savvidou et al., 2003, p. 395) served to threaten parents’ sense of acceptance and safety in the communities they lived within. Parents demonstrated pervasive self-defeating perceptions about their parenting competence, which appeared to be exacerbated by idealised societal perceptions of parenting, threatening their sense of security in their parenting roles. Negative self-perceptions caused parents to lack parenting confidence, which appeared to trigger feelings of hopelessness: “*I’m never going to be able to be the person I’m meant to be to raise them*” (Perera et al., 2014, p. 176). Parents reported feeling alienated from parenting peers, choosing to avoid parenting networks, bound by fears of negative social consequences.

*“If other mothers knew I had a mental illness, they might not allow their children to play with mine”* (Diaz-Caneja & Johnson, 2004, p. 477).

Parental fears were, however, a reality for some, with potent stigma permeating across generations: *“…She [child] said their mothers told them not to play with her because her mother was crazy*” (Rampou et al., 2015, p. 124). Consequent of parental fears of rejection, parents’ authentic selves remained hidden, bound by a powerful desire to be seen as “*ordinary people*” (Tjoflåt & Ramvi, 2013, p. 88).

### Subtheme 4.3: Inappropriate Support

Across studies, parents largely reported feeling alone without the support of systems around them: “*It may be important that you know that sometimes the structure around us fails*” (Strand et al., 2020, p. 627). Parenting status was perceived to be largely unrecognised by HCPs, serving to undermine parental trust in service provision and contributing to parental threat perceptions. Furthermore, parents whose needs did not fit precise service entry criteria remained unsupported and vulnerable to the powers of services that they hoped would support them.

*“…Then they would say ‘Your case does not fit,’ why should I keep trying?*” (Chan et al., 2019, p. 532).

Often HCPs and family expressions of fear about child safety, based on associations with diagnostic labels, could result in increased parental perceptions of being observed, as reported in several studies (Chan et al., 2019; Khalifeh et al., 2009; Montgomery et al., 2011; Perera et al., 2014; Wilson & Crowe, 2009). This risk-focused approach threatened parents’ sense of control, exacerbating perceptions of powerlessness, threat, and inadequacy which could distance parents from accessing support. Amongst parents living within services, significant threats to the integrity of parent–child relationships were posed by the combination of child access limitations and inappropriate visiting facilities: *“The hospital is not the right environment for them*” (Diaz-Caneja & Johnson, 2004, p. 478). Although respite associated with inpatient care was a welcome relief for some, many parents believed the support received did not adequately prepare them to return to parenting at home, leaving them feeling unprepared and anxious.

Wider socio-economic factors further threatened parents’ sense of security. Reliance on other people for financial support was degrading experiences. Several studies (Ackerson, 2003; Chan et al., 2019; Rampou et al., 2015; Sabella et al., 2022) reported that the systems around parents and their children could threaten their basic human needs, for example, for connection and emotional safety.

### Theme 5: Combatting Threat: Holding Hopes, Goals, and Aspirations

This theme was present in 19 of the 29 included studies. Parents were largely able to sustain their parenting roles, adopting “*small tricks*” (Tjoflåt & Ramvi, 2013, p. 87) to combat the challenges presented to them. System-wide support helped parents to manage their difficulties, whilst having hope, aspirations, and parenting goals supported parents to reduce the impact of threat to support their parenting. Many parents reported finding solace, pride, and comfort in their parenting roles, with children enriching their lives and promoting a sense of hope, *“…sort of quite life affirming. It jogs me out of the depression that used to sort of get me down*” (Evenson et al., 2008, p. 637). In addition, children were often perceived to offer hope of meeting parents’ relational needs for reciprocated love: *“I felt that I loved this little person completely, and this little person would love me*” (Diaz-Caneja & Johnson, 2004, p. 475).

Parents who reported feeling hopeful and optimistic for their future lives with their children demonstrated increased commitment to change. This finding was particularly evident in parents who had been separated from their children and who were supported to re-gain contact and parental responsibility. The integrity of the parent–child relationship was highly regarded and considered a priority goal. Themes of aspiring for security and comfort prevailed across parental goals and aspirations, offering parents a sense of optimism and hope for the future: “*I want to get stable. You know, get settled in my relationship with [my son]…*” (Mulvey et al., 2021, p. 20).

### Theme 6: Wrap-Around Support Needs

Parents and children were situated within complex systems spanning family, peer, and wider socio-political contexts, captured in two sub-themes. It was clear that those who parented without support experienced the most significant challenges and some considered it unrealistic to raise a child alone: “*I believe it takes a village to raise a child*” (Ackerson, 2003, p. 116). Parents considered it crucial for services to recognise and provide early, multi-disciplinary and system-wide support:

*“Mental health professionals and the children and family social services department have to be more incorporated. They have to become more of a joint body and have some kind of co-ordination and co-operation going fully”* (Diaz-Caneja & Johnson, 2004, p. 479).

### Subtheme 6.1: System-Wide Compassion and Understanding

A fundamental need to trust, be understood by, and connected to family, peer, and professional systems was reported by many parents across studies. Whilst HCP support was variable, parents valued respectful and consistent approaches within which they felt understood “*without judgments*” (Montgomery et al., 2006, p. 25). A compassionate approach appeared to be necessary in supporting parents to feel empowered and understood; an important step in targeting power differentials that often underpinned barriers to parents accessing support. Relatedly, parents wished to receive support from people who they felt would understand their position due to their lived experience, both from HCPs, “*I wanted a mum as a GP…”* (Awram et al., 2017, p. 155), and peer support groups:

“*If you talked in a group setting with other parents, where they understood what psychosis was, and you could share different experiences, and then maybe share things that have worked, and then also it’s then sociable as well, and you may gain, sort of, friends out of it*” (Radley et al., 2022a, p. 8).

Parents hoped such support would allow them to feel “*less burdened*” whilst simultaneously promoting parenting support by helping parents “*learn some lessons from other people*” (Chen et al., 2021b, p. 7).

Furthermore, although guilt and shame often limited parents from feeling able to make time to meet their own needs, parents who reported feeling empowered to consider their own needs experienced richer connections both with themselves and their children.

*“So once I learnt that, that made a huge...like light bulb moment so that I knew ‘ok if I start looking after me and my mental health and my physical health then I’ll be able to look after my family”* (Awram et al., 2017, p. 152).

The need for psychoeducation for parents, children, and their families, alongside wider peer and professional networks was frequently reflected as necessary to promote inclusion and connection and reduce blame, stigma, and fear. Parents wished to understand their symptoms: “*I want to know more about bipolar…why I become irritable like this*” (Rampou et al., 2015, p. 0124). With such an understanding, parents could be afforded more control over their parenting, with alternative parenting strategies becoming more comprehensible and accessible. Support from mental healthcare professionals that specifically targeted parenting difficulties was regarded as important for some in alleviating distress and potential adverse outcomes for children. Practical advice and information were sought about how to approach specific parenting circumstances, including balancing control and managing emotions.

*“Let’s say I get angry at my child...how can I manage that with my child? Or is it okay if I don’t deal with it? If I need to deal with it, then what should I do?”* (Chen et al., 2021b, p. 7).

The role that psychoeducation could offer for children was particularly welcomed for parents who worried that children would “*blame themselves*” (Chen et al., 2021b, p. 7) for parental emotional difficulties. Healthcare professionals, including nurses, were considered to be professionals that could play an important role in supporting family-wide understanding and engagement via psychoeducation: “*It would be nice if nurses talked about the transference of psychiatric problems to the children*” (van der Ende et al., 2016, p. 90).

### Subtheme 6.2: Connection to Support

A dominant theme across studies was the need for parents to feel able to rely on systems around them to meet their children’s needs when unable to do so alone: *“…If only there was someone there to help me look after my children…I could only try my best to stay at home and control myself*” (Chan et al., 2019, p. 532). Parents who were part of supportive family networks reported having an additional “*backbone*” (Tjoflåt & Ramvi, 2013, p. 84), providing additional strength to support their parenting. Parents placed significant weight on remaining the primary carer of their children during acute periods of psychological distress and believed that separations during inpatient stays could be avoided. There was a sense that mothers in particular felt bound by the powers of child custody authorities but wished that services would support children remaining with them during inpatient stays.

*“If it was possible that when you are admitted at the hospital and your child doesn’t have anybody to take care of him/her, they should allow us to sleep with them in the hospital until we are discharged”* (Rampou et al., 2015, p. 124).

Parents valued being close with their children, but also needed space for self-care. The idea of family-focused support, within which both parents and their children could be simultaneously supported both therapeutically and socially, was considered a valuable system-wide intervention. Parents considered it important for services to consider both their own mental health needs and how their children might need to be supported in relation to their mental health needs. Parents reflected that services that supported respite care for children would be helpful in promoting space for parental self-care.

*“I think there needs to be like a place where we could take our kids to take them somewhere because we need time to ourselves but I mean for just bipolar, you know”* (Venkataraman & Ackerson, 2008, p. 404).

Across studies, parents reflected that support should be extended to their children; it was not enough for parents to receive support alone.

*“As much as I have to go to a psychiatrist or a psychologist and chat, the kids have to be allowed to go...they’ve got so many thoughts in their heads*” (Klausen et al., 2016, p. 112).

## Discussion

This systematic review of 29 studies is the first to comprehensively synthesise qualitative research exploring mothers and fathers’ experiences and perceptions of the impact of SMI on parenting and their support needs without being restricted to specific cultures or specific mental health difficulties within the SMI umbrella. Key themes were identified regarding the challenges that parents who experience SMI are faced with, factors that contribute to and maintain parenting difficulties, parental coping strategies, and parental support needs. Findings from previous reviews and studies regarding the interplay between parental perceptions of inescapable, system-wide threat, and parents’ current and desired use of family, peer, and professional support have been extended within the current review.

The current review consolidates and extends findings featured in previous reviews of mothers who experience SMI (Dolman et al., 2013) and parents with diagnoses of bipolar disorder (Stapp et al., 2020) and significantly enhances findings regarding the centrality of parenting difficulties in the lives of parents who experience SMI and the aversive impact of stigma and fears of child loss on parenting relationships. The current review extends findings by reporting on the experiences of mothers and fathers across cultures, childcare, and living contexts. Across contexts, parenting challenges and relationships appeared situated within complex systems underpinned by persistent threat, further compounding SMI-related parenting challenges, regardless of parent gender and living arrangements.

Novel insights are presented into how factors including inescapable perceptions of threat and unstable parental identity perceptions can impact the polarisation of parent–child relationships and role reversals; a finding that augments recent research highlighting that children can perceive themselves as parenting figures when supporting parental SMI (Villatte et al., 2022). The influential role of systemic threat was clearly communicated throughout participants’ narratives, in which systems that parents perceived to neglect and threaten their parenting identities exacerbated their difficulties and fears. In turn, strain was placed on parent–child relationships which increased parental feelings of guilt and shame, resulting in distance between parents, their children and the systems around them. These findings support previous reports highlighting the central role of power, threat, and deficiency of sense making amongst people who experience psychosocial distress (Johnstone & Boyle, 2018).

Previous research has highlighted factors that inhibit parents from accessing support, including lack of policy and practice guidelines, lack of integration between adult and child services, crisis-orientated service provision, fears about child loss, and approaches that present a parenting ‘fix’ (Jones et al., 2016; Mulligan et al., 2020, 2021; Tuck et al., 2022; van Esch & de Haan, 2017). The current review consolidates such findings and offers insights into how to target such barriers, by moving away from siloed and risk-focused approaches in which practitioners and policymakers are at the centre of decisions, and towards a system in which practitioners and other stakeholders scaffold compassionate, goal and strength based and collaborative support around parents, and the systems they live within. Our findings suggest that policy guidance underpinning clinical practice may inhibit professionals from providing the type of care that would allow parents to meaningfully engage with services, as explored recently by Tuck et al. (2022). Echoing Bronfenbrenner’s *Ecological Systems Theory* (Bronfenbrenner, 1992) that views psychosocial processes to be influenced by multiple levels of the surrounding environment, our study findings highlight that change is needed at multiple systemic levels to promote better relationships between parents and their children, families, HCPs, and wider cultural and political networks. In line with recent calls for a ‘village’ approach of social connectedness for families who experience multiple adversities (Goodyear et al., 2022; Reupert et al., 2022), a shift in practice approach is indicated, particularly given that Family Focused Practice (FFP) is not widely implemented even within countries that mandate it (Falkov et al., 2016; Furlong et al., 2021). A strengths-based approach could shift system-wide perceptions of threat, promoting parents’ sense of safety and connection with their children, their parenting roles, and the communities they live within, supporting better access to, and use of, support. In turn, this could increase parent–child and system communication and connectedness, for example, by targeting the well-referenced barrier of stigma (Lacey et al., 2015).

### Clinical Implications

The current review highlights key aspects relevant to the successful implementation of evidence-based policy and practice that are grounded in qualitative data and driven by the voice of parents (Skivington et al., 2021). The present review should prompt parents, practitioners, commissioners, and policymakers to consider the implications for practice, in line with a systems’ approach that places parenting support in a wider systemic context (Allchin et al., 2022; Bauer et al., 2021; Falkov et al., 2016; Mytton et al., 2014). A strengths-based system-wide FFP approach is indicated, putting families at the centre of support decisions, promoting layers of support around parents, and decreasing social adversity and threat; a factor reported to be more detrimental to child outcomes than SMI itself (Gladstone et al., 2011). A system-wide FFP approach has the potential to decrease risk of adverse outcomes for parents and children, reduce referrals to child protection services and the need for reactive and crisis-based interventions (Nicholson et al., 2019), and promote better communication and connection between parents, their children, and the systems they live within. Recommendations based on parents’ reported experiences and support needs are provided in Table 5. However, given that the current review did not include studies reporting on the views of children, HCPs, or commissioners, caution should be given when considering these recommendations.

**Table 5:** Suggested clinical implications and recommendations

Area of the system	Recommendation
Parents	• Psychoeducation should be provided to normalise parents’ experiences, reduce guilt and stigma, promote integration with community networks, support system-wide conversations about mental health, and support parent–child attachment relationships • Parents should be supported to access peer networks to tackle parental isolation • Parents’ psychological and practical support needs should be considered from an early stage to avoid crisis escalation and restrictive interventions. A strengths-based approach could support parental hope and goal-based parenting outcomes • Longer-term psychological support could support parents to make sense of experiences of threat, supporting parents to re-gain their sense of control and connection with their children, families, and wider networks • Emotion regulation support should be considered, if necessary, to support parent–child relationships by reducing shame and self-defeating behaviours by supporting parental recognition and management of their own and their children’s emotional needs • Practical parenting support and respite care should be considered, particularly for parents without system supports. Practical factors should be considered, including childcare provision and flexible service access arrangements
Children and family	• Consideration should be given to providing respite care for children, particularly children who have been identified as experiencing increased responsibility to care for their parent(s). Community support groups could also provide connection and containment for these children and other family members • Child and family well-being should be monitored to promote signposting and joined up support to appropriate healthcare and community services • Psychological support for children should be considered, providing opportunities for safe and supportive exploration, sense making, and management of psychosocial difficulties
Healthcare services	• Specialist training, support, and supervision should be offered across parent and child services to ensure that necessary knowledge, skills, confidence, competence, and compassion underpins service delivery. This could help reduce practitioner fear- and risk-orientated responses, in turn fostering parental hope and trust in services • HCPs should hold in mind the centrality of parenting identity in the lives of people who experience SMI. Parenting status should be asked about and considered by all HCPs • Consideration should be given to socio-cultural and political contexts within which parents live, promoting a cultural fit of service delivery • Sensitivity to parental distress and fears of social service involvement is required. Services should address parental concerns to alleviate fears and promote engagement
Policy and legislation	• Services and communities should be adequately funded to ensure suitable provision of staff, training, and resources to meet parents needs as outlined above • Public awareness of experiences of SMI should be increased to target stigma and promote non-judgmental, compassionate, and connected system-wide support • Socio-economic disadvantage, adversity, and wider systemic influences should be accounted for

### Strengths, Limitations, and Future Research

A comprehensive systematic search was conducted, and data were synthesised from 29 studies reflecting the voices of 562 parents experiencing SMI across 14 countries, spanning 27 years of research. A range of childcare, living arrangements, and socio-cultural factors were represented within parent samples, allowing for the analysis and interpretation of a diverse range of parental views and experiences, representing a strength of the review. However, only 38% of the included studies reported on ethnicity, and studies were included that were based on both inpatient and outpatient settings, which limits the transferability of findings across ethnic groups and settings. Future research should explore and report on ethnicity, especially given the higher prevalence and poorer outcomes for people experiencing mental health difficulties amongst ethnic minority groups (Maura & Weisman de Mamani, 2017). Another future study should explore the experiences and needs of parents within specific healthcare settings, including perhaps inpatient settings only.

Although it was not possible to explore specific parental experiences and specific mental health experiences were deliberately not explored, it was necessary to first establish this comprehensive and broader understanding of the impact of parental SMI and support needs to guide future research, policy, and interventions. The current review was restricted to peer-reviewed studies published in English or German as the research team was fluent in these languages and due to time limitations and translation costs. Language, publication, and selection biases are therefore possible, and caution is advised when generalising findings. Future research should explore parental barriers to accessing services across specific geographical locations, cultures, and settings and should include the views of families, HCPs and policy makers. Further, given that adoptive, foster, and kinship parents were not reported within the included studies, future research should explore their experiences and needs. Although it is not possible to transfer findings from the contexts of the included studies in this review to geographical regions, cultures or specific mental health difficulties, clear themes emerged from the data irrespective of setting, location, and mental health difficulty, highlighting key recommendations for practice and future research.

The use of thematic synthesis allowed multiple qualitative approaches and findings to be synthesised, promoting new interpretations to inform policy and practice. The themes derived from the synthesised data are acknowledged to be influenced by researcher-lived experience, position, and insights, and the analysis relied on author interpretations and illustrative quotes chosen by the authors. However, the trustworthiness, methodological rigour, and credibility of the review findings were enhanced through the process of independent review at stages of study selection, quality assessment, and theme identification (Tong et al., 2012) and due to the high or moderately high methodological quality ratings of all included studies.

## Conclusion

This was the first review to comprehensively synthesise qualitative research exploring mothers’ and fathers’ experiences regarding the impact of SMI on parenting and their support needs that was not restricted by specific cultural characteristics. Parental perceptions of inescapable threat profoundly impacted parent–child relationships, which were strained and centred around SMI-related parenting difficulties. The need for system-wide support placing parenting in a compassionate systemic context is emphasised. Key recommendations for clinicians and policy makers are highlighted. Future research should consider the experiences and support needs of parents with specific parenting and mental health challenges.

## Supplementary Information

Below is the link to the electronic supplementary material.

Supplementary file1 (DOCX 22 kb)

Supplementary file2 (DOCX 23 kb)

## Data Availability

The data that support the findings of this systematic review are available from the corresponding author upon reasonable request.

## Footnotes

1 The authors of this paper prefer to use the term mental health difficulties. However, the term SMI has been used throughout this paper due to it being a widely recognised term in the wider literature.

## Declarations

### Standards of Reporting

This metasynthesis was conducted in line with PRISMA guidelines (Moher et al., 2009).

### Secondary Analysis of Existing Data

This study is a re-analysis of existing data that are publicly available. This study brought together existing research data obtained upon request and subject to licence restrictions from a number of different sources.
