# Supplementary material for: Parenting and Serious Mental Illness (SMI): A Systematic Review and Metasynthesis
Source: Clin Child Fam Psychol Rev. 2023 Feb 18;26(2):303–42. doi: 10.1007/s10567-023-00427-6 (PMC10123049; doi:10.1007/s10567-023-00427-6)
Supplement: Supplementary file 1 — Supplementary file1 (DOCX 22 kb) [file 10567_2023_427_MOESM1_ESM.docx]

***Supplementary Material 2. Table of the Six Main Themes, 13 Subthemes and Additional Exemplar Quotes***

| **Theme** | **Subtheme** | **Additional Exemplar Quotes** |
| --- | --- | --- |
| 1. The Constrained Parent | - 1. ‘Perfect’ Parenting Standards | ‘I was possibly asking myself to be more perfect [as a mother]. I couldn’t accept that I wasn’t able to be extra good to her [the child] with all my heart and soul.’ (Chen et al., 2021).  “You never tell people you have got bipolar disorder, because they think you are nuts, that you go completely Jesus Christ, run up and down the street naked” (Wilson & Crowe, 2009).  “I had a long time in those early days when I felt really useless as a parent, really, really useless” (Wilson & Crowe, 2009). |
|  | - 1. Emotion Regulation Difficulties | “I would get really angry. There was so much going on in my head, and it was so loud and I didn’t know that wasn’t normal, and it would make me snap and get angry and just everything seemed to be coming at me at once. Um, and that. . . being a mom, it was hard cause I would feel so bad, I would yell at my kids or you know, just get frustrated and I wouldn’t be able to focus. . .cause there was so much thought going on. . .it [her anxiety] definitely affected me in a lot of ways and it was just overwhelming I think” (Mulvey et al., 2021).  “I would hit him. It served him right…I didn’t know why I could not stop…” (Chan et al., 2019)  “I feel guilty a lot of the time because I get irritable with them, I get impatient and I don’t know whether that is my illness or whether that is normal at times, it is hard to work out” (Wilson & Crowe, 2009). |
|  | - 1. Fears of Repeating History | “I have a great fear that they too will suffer from mental illness, either genetically (my mother also had mental health problems) or socially because of what they have been through” (Diaz-Caneja & Johnson, 2004).  “I get reminded of when I was a little, and I don’t want him to experience the same… He gets sad when we play games and he loses…so I let him get what he wants” (Strand et al., 2020). |
|  | - 1. Avoidance and Masking | “So you hide I – you try to be a good parent, but you hide it when you are not well...” (Wilson & Crowe, 2009).  “I feel like with child welfare authorities that they really. . .like follow me with their eyes, how I am, how I seem to be...if I have a bad or good day, so I try-yes, to feel good…” (Klausen et al., 2016).  “I’m trying to give them as normal a life as possible and you know, I would rather keep it to myself and not worry them if I don’t need to worry them” (Radley et al., 2022). |
| Parenting Difficulties | - 1. Struggling for Control | “I was not good at saying no, because I felt sorry for them [because they had been through so much] ... I tried to make their life easier” (Ackerson, 2003).  “I found that there were no boundaries. . . . I didn’t feel like cooking, so what I would do is spend money that I didn’t have, and just give them money to buy takeaways and things like that, and letting them play out[side] when normally I would put my foot down” (Khalifeh et al., 2009). |
|  | - 1. Balancing Needs | “It is hard to do anything being a full-time mother, you are running around after them all the time, cleaning up and you don’t have time for yourself” (Perera et al., 2014).  “There was a point where I just managed, and there was a point where I knew I couldn’t do it anymore. I couldn’t run the whole struggle, not even look after myself, much less to look after a child.” (Khalifeh et al., 2009). |
|  | - 1. Amplification of Struggle due to Symptoms and Medication | “. . .when I have those slips of memory, which are part of my illness, something major will get lost . . . and when it relates to the kids, that makes it hard to be a dad. I feel ashamed of having fallen short of my standards . . .” (Evenson et al., 2008).  “Oh, they love it. They love it because we are at the park every- day, and I go around like animal with them, and then, you know, just going up and down the slide, you know, they love it, because mommy is so happy, you know, hey, if mommy has got all this energy, that most parents don’t have, you know…” (Venkataraman & Ackerson, 2008).  “For me, it’s sad, but it makes it harder for me to put my child first. And makes it harder for me to focus more on his well-being than what I want to do for myself. Which sounds really bad. It sounds really sad. But it’s like if you want the honest to God truth, that’s the honest to God truth. And I wish that it would be better. And I wish that I could put more focus on to him in making sure that he’s good before anything else. But sometimes it’s just like my brain won’t let me do it. It’s just like I come first. Which I don’t want to. He’s my baby” (Sabella et al., 2022). |
|  | - 1. Connection to Child | “when you are mentally unwell, you don’t spend as much time with your child because you are so consumed by your mental health” (Perera et al., 2014).  “It’s difficult when my son gets anxious, I think. It’s hard not to go in and mix it up with myself, to project my feelings on him. It’s hard to see him as an individual...” (Strand et al., 2020). |
| 1. The Strained Child: Role Reversal and the Perceived Impact | | “I think he carries many hard things within himself that he doesn’t talk to me about. He talks to my care provider about it, and he doesn’t say it to me. He doesn’t want to hurt me, he wants to protect me and not make me sad” (Strand et al., 2020).  “She became the little parent sometimes, and because she had that little bit of responsibility or something, it made it even worse for me to parent her!” (Ackerson, 2003).  “Your mother is a patient. I don’t have the energy to discipline you ... So, you better manage yourself”, (Chen et al., 2021).  “If you're feeling not too good in yourself, it seems like the kids, they sense it. And then they act out more” (Nicholson et al., 1998).  "T [Son] then withdrew completely and didn't come any more. And A. [daughter] was overwhelmed and cut herself and all sorts of things. And then somehow during the follow-up consultation with the child psychiatrist she just said that she couldn't stand it with me any longer” (Jungbauer et al., 2010). |
| Inescapable Threat | - 1. Loss and Separations Fears | “If I did anything that made them think I was going crazy then they’d take my daughter away” (Diaz-Caneja & Johnson, 2004).  “I think I would die if my daughter was every taken away from me, especially for the wrong reasons - you know, incompetence” (Nicholson et al., 1998).  “I always thought they wanted to take my kids away from me. I saw everything in a negative way” (Jungbauer et al., 2011). |
|  | - 1. Stigma and Fears of Rejection | “It is quite hard to ask for help when you need it because everybody thinks, oh she’s a bad mother, she can’t do this . . .” (Diaz-Caneja & Johnson, 2004). |
|  | - 1. Inappropriate Support | “Before my daughter was born I went to therapy and this information was passed on to Child and Youth Care. It [the information] followed its own course. Instead of being supportive, they kept me under strict control, based on the psychiatric diagnosis in my file” (van der Ende et al., 2016).  “I was a bit worried about what they [children] might see, a lot of people wander around in a daze and look a bit nutty ... I was worried that they might think I was like that too” (Tjoflåt & Ramvi, 2013).  “I’ve definitely been discriminated against by the judge. It was horrible. They’d talk to me like I was five. They looked at me like I was a disease of a father. I wanted to be there for my kid but was never given the opportunity by the courts to do it” (Sabella et al., 2022).  “I’ve not got that support there so if it happens again I can’t just go and phone someone and say, ‘come and get him I’m not feeling very well’, because I know no one would come” (Radley et al., 2022). |
| 1. Combatting Threat: Holding Hope, Goals and Aspirations | | “I want them to grow up in a decent, pleasant, lovable environment that doesn't have any fighting, arguments, you know any kinda physical abuse. I want to have a nice home where I can own some day…I'd like to just live the rest of my life out with my kids in peace…” (Sands, 1995).  “I want to get stable. You know, get settled in my relationship with [my son]. . .I mean, like re-establish. . .Slowly, confidently, you know, so he can be confident that I’m not gonna get, you know [crazy] again” (Mulvey et al., 2021). |
| 1. Wrap-around Support Needs | - 1. System-wide Compassion and Understanding | “My mother saw that I was isolating myself, that my world became small and that I got stressed by raising my child. She said to leave him [her child] with her so I could breathe” (van der Ende et al., 2016).  “…Having a work setting that accommodates day care, parental concerns, makes a big difference” (Ackerson, 2003).  “I need a GP. I wanted a mum as a GP ... Cos I would say to her, “Is this a normal mother problem or is this a crazy mother problem?”’ (Awram et al., 2017).  “I had a serious diagnosis, but I didn’t understand it, and perhaps neither did my therapists or my doctor. I was alone with my children, I was manic and psychotic and we didn’t get any help. Had they understood it and had my children been in kindergarten, it would have helped, both for my children and me . . . it’s quite as simple as that” (Tjoflåt & Ramvi, 2013).  “It would be nice if nurses talked about the transference of psychiatric problems to the children. They should make it clear that we have to deal with it . . . although it is hard to do it.” (van der Ende et al., 2016). |
|  | - 1. Connection to Support | ‘‘I think there needs to be like a place where we could take our kids to take them somewhere because we need time to ourselves but I mean for just bipolar, you know” (Venkataraman & Ackerson, 2008).  “I think it would be a group of people meeting up regularly, and perhaps the opportunity for their children to be there. Somewhere where you could talk, where you could have outings as well as get families together. And support from professional people there, not just people talking, but professional people helping them to cope as well…” (Diaz-Caneja & Johnson, 2004). |
